# Node Modification of Metal‐Organic Frameworks for Catalytic Applications

**DOI:** 10.1002/open.202400428

**Published:** 2025-02-26

**Authors:** Mario Martos, Isidro M. Pastor

**Affiliations:** ^1^ Organic Chemistry Department and Institute of Organic Synthesis (ISO) University of Alicante, Ctra. San Vicente del Raspeig s/n, San Vicente del Raspeig Alicante 03690 Spain; ^2^ Department of Chemistry and Molecular Biology University of Gothenburg, Medicinaregatan 7B 413 90 Gothenburg Sweden

**Keywords:** Metal-organic framework, Post-synthetic modification, Node, Cluster, Catalysis

## Abstract

Metal‐organic frameworks (MOFs) have been a breakthrough in different fields of chemistry, not only due to the extensive possibilities regarding their synthesis, but also the ease of modulation of the structure's properties by chemical modification of linkers and nodes. The latter is particularly interesting in heterogeneous catalysis, as the newly inserted species may enhance, regulate, or straight enable new forms of catalysis unattainable by the pristine material. This acts in conjunction with the parent MOFs providing selectivity (e. g., by size exclusion) and protecting highly reactive catalytic species, offering increased stability and robustness to well‐known catalytic systems. In this review, we compile the most relevant post‐synthetic modification of the nodes of well‐known MOFs of the last decade (2015–2024) and their application to heterogeneous catalysis. This review is divided into two main sections covering modifications involving metallic species and organic moieties, with sub‐sections for each MOF on both. This way, we aim to provide a broad view of the state of the art while showcasing the expanded catalytic properties of the resulting materials.

## Graphical abstract

Post‐synthetic modification of metal‐organic framework (MOF) nodes with metal or organic species produces advanced materials that serve as efficient catalysts, promoting more sustainable chemical transformations.

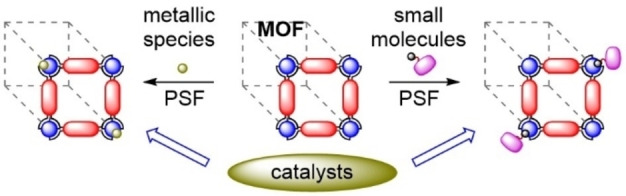

## 1 Introduction

The term “Metal‐Organic Frameworks” (MOF), coined by Omar M. Yaghi in 1995,^[1]^ refers to a series of hybrid materials constituted by metallic centers bridged by organic molecules forming two‐ or three‐dimensional coordination networks with permanent porosity.^[2]^ Metal nodes are constituted by lone ions, with simple connectivity, or polynuclear inorganic clusters, with multiple coordination positions.^[3]^ These structures, named secondary building units (SBUs),^[4]^ are the core components of the MOF and their geometry generally dictates the final topology of the material.^[5]^ More than 130 SBUs of different metals are known, with vacant coordination positions ranging from three to sixty‐six.^[6]^ Regarding organic linkers, they are molecules bearing at least two chelating groups and having a certain degree of rigidity.^[2]^ Although carboxylates are the most universal chelating moieties, other groups, such as alcohols, amines, phosphates or nitriles have also been employed. An adequate selection of both linker and metal is important, as it plays a large role in the stability of the resulting material, which is one of the main drawbacks of MOFs compared to other materials when targeting industrial applications. Chemical stability can be largely rationalized following the hard‐soft acid‐base (HSAB) theory proposed by Pearson, which dictates that hard cations (high valence transition metals such as Ti^4+^ or Zr^4+^) are best paired with linkers such as carboxylates, whereas soft metals (e. g. Cu^2+^, Zn^2+^) should be matched with soft ligands such as azolates. Of course, this is not enough to fully determine how stable a given structure is, as there are additional factors in play. Against water, the stability of the material is governed by thermodynamic (metal‐linker bond strength, redox potential of the metal) and kinetic factors (e. g. hydrophobicity of the structure, steric shielding of the nodes). For further reference, the reader is directed towards two excellent reviews on the design of stable MOFs and a comprehensive analysis of the behavior of well‐known structures against water.[^7^, ^8^]

On the topic of mechanical stability, a certain level of rigidity is essential in obtaining materials with permanent porosity, which differentiates regular coordination polymers (CPs), known since the 1950s, from MOFs, which have their metal centers conformationally locked in place, ensuring the rigidity of the framework.[^2^, ^9^] MOF‐5, formed by ZnO_4_(CO_2_)_6_ SBUs and BDC (benzene‐1,4‐dicarboxylate) linkers, represents the first reported metal‐organic coordination network with permanent porosity.^[10]^ A special kind of non‐SBU‐containing MOFs are zeolitic imidazolate frameworks (ZIF), formed by Co(II) or Zn(II) ions bonded by imidazolate linkers. These materials have very stable structures due to their short and completely rigid linkers, as well as the high bond strength between the components.^[6]^ MOFs exhibit exceptional modularity based on the virtually endless combinations of metal nodes and linkers available, including the possibility of forming isoreticular MOFs from elongated linkers.^[9]^ This modularity allows the preparation of highly specialized materials, which offer a series of advantages over reference porous materials such as zeolites, silicas and activated carbons for certain applications.^[2]^ Even within the same MOF structure, it is possible to control parameters such as particle size, crystallinity and defect density through the use of modulators, i. e. monodentate ligands bearing the same chelating group as the linker.^[11]^

The vast majority of MOFs are obtained via solvothermal processes, in which solutions of the components (commonly in polar solvents with high boiling points, such dimethylformamide or water) are combined and subjected to vigorous heating for extended periods of time.^[12]^ The MOF precipitates out of the solution as a somewhat amorphous powder due to fast nucleation, which rearranges under solvothermal conditions to a more crystalline material.^[8]^ However, this process is slow, requiring high temperatures and enough time, and there is little to no control over the particle size or defect density of the material.[^11^, ^12^] In modulated syntheses, the SBUs are initially formed with the modulator, which is added in excess. Then, the MOF is formed by displacement of the modulator by the linkers. Although the kinetics of the process are determined by temperature and the modulator/linker ratio, they are inherently slower, thus affording a more crystalline material.[^12^, ^13^] An interesting implication of the mechanism of action of modulators is that, as the initial SBUs are occupied by modulator molecules rather than linker, control over the defect density of the final material is attained by simply adjusting the reaction time.[^14^, ^15^] Then, modulators attached to the clusters may be removed, providing materials with catalytically active free sites, or kept in place, obtaining node‐functionalized materials. With over 95000 different structures compiled at the Cambridge Structure Database as of 2020,^[16]^ MOFs are regarded by many as the greatest advance in the field of porous materials of the last 30 years. Their modularity has allowed for the development of highly task‐specific materials for application in selective gas absorption and storage, drug delivery, as solid electrolytes, molecular sieves, and templates for hybrid and inorganic materials.[^2^, ^17^, ^18^, ^19^, ^20^, ^21^, ^22^, ^23^, ^24^, ^25^, ^26^, ^27^] Heterogeneous catalysis deserves a mention of its own. Besides the general advantages of supported materials (e. g. increased stability of sensitive catalytic species, recoverability), MOFs offer the possibility of controlling the structure and hydrophobicity of the framework, which enables further control of the reactivity (e. g. by size exclusion or selective adsorption of reactants). Beyond the ample variety of possible building blocks and controlled formation techniques, post‐synthetic modification (PSM) methodologies offer additional control over the physical‐chemical properties of MOFs and further extend their applicability.[^2^, ^12^]

## 2 Post‐Synthetic Modification of MOFs

**Figure 1:**
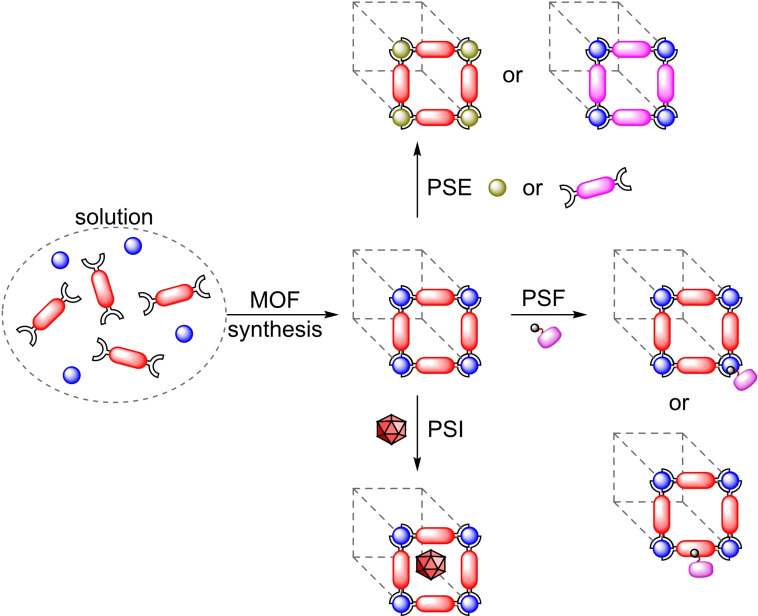
Post‐synthetic modifications: PSE (post‐synthetic exchange); PSF (post‐synthetic functionalization); PSI (post‐synthetic insertion).

The post‐synthetic modification protocols for MOFs are divided in three categories, namely post‐synthetic exchange (PSE), post‐synthetic insertion (PSI), and post‐synthetic functionalization (PSF) (Figure 1).[^8^, ^15^] PSE involves exchanging metals or linkers within the original framework for structurally equivalent replacements, resulting in hybrid or fully exchanged MOFs impossible to obtain through normal means. This process is commonly achieved by immersing the structure in a solution (usually a polar solvent capable to stabilize the unsaturated metal intermediates) containing excess amount of the intended replacement components. Regarding PSI, it involves the encapsulation of catalytically active species within the structure of the MOF. The PSI of organic compounds and metal complexes by diffusion of the precursors into the pores of the MOF is known, although there is the possibility of adding the guest molecule to the growth media of the MOF, known as the “bottle‐around‐a‐ship” approach. PSF consists in the functionalization of the MOF components by means of heterogeneous‐phase reactions and is one of the most prevalent forms of modification, particularly regarding linkers. Linker PSF involves a transformation of a functionalized unit, which may have been directly used to synthesize the MOF or inserted via PSE. The modification of metal nodes is not as prevalent, due to its inherently higher complexity. Both organic functionalities and metallic species may be used to modify the node. The former are usually introduced by submerging the material in a solution of the molecule of interest, which should bear at least one chelating group to coordinate to the node. Due to the conditions usually employed, this approach is named SALI (solvent‐assisted ligand incorporation). Metallic modifications can too be introduced by solvothermal immersion (SIM) protocols, which are analogous to SALI, although the grafting of the metal usually takes place at the hydroxyl groups of the nodes. An additional technique is available for metal modifications. Atomic layer deposition (ALD), consisting in the reaction of the nodes with metallic precursors in vapor phase, allows for higher precision in the distribution than SIM, although it does require specialized equipment and is more operationally complex.

In this revision, the most relevant advances of the last decade in node modification of metal‐organic frameworks to obtain catalytic materials have been compiled, aiming towards elaborating a quick but comprehensive reference guide for the MOF chemist. Two main categories have been considered: (a) node modification by metallic species; (b) node modification by organic molecules. For other types of modification, such as nanoparticle encapsulation^[28]^ as well as in‐depth revisions of MOF‐based catalysis,[^3^, ^29^, ^30^] the reader is directed towards some excellent reviews on the matter.

## 3 Modification of Nodes with Metallic Species

Different metallic species have been used to modify the catalytic properties of well‐defined metal‐organic frameworks, obtaining hybrid materials which have been proven as effective catalysts in a plethora of synthetic transformations, some of which inaccessible to the pristine MOFs. The examples gathered herein are discussed in the following subsections.

### 3.1 NU‐1000

**Figure 2:**
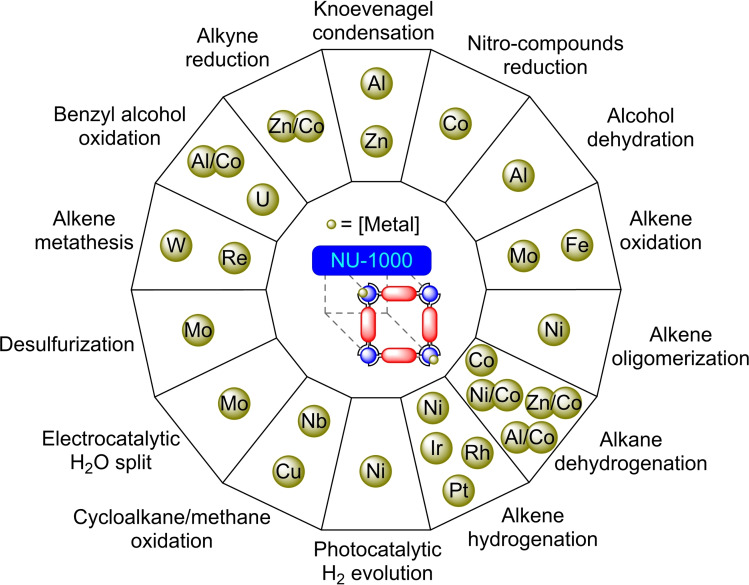
Post‐synthetic modifications of NU‐1000 with metals and their applications.

Among the zirconium‐based metal‐organic frameworks, NU‐1000 has a high surface area and exhibits good chemical and thermal stability. NU‐1000 is prepared employing 1,3,6,8‐tetrakis(4‐benzoate)pyrene linkers. This material has mesoporous (31 Å) and microporous (12 Å) channels. In addition, NU‐1000 nodes have hydroxyl (−OH) residues, which are terminal and point to the channels, allowing coordination of other metal centers. Its characteristics, together with the possibility of an easy and scalable synthesis, have enabled extensive studies in the field of catalysis.^[31]^ The nodes of this MOF have been modified with a wide variety of metals, which have allowed oxidation, reduction, condensation, or polymerization processes to be carried out, among others (Figure 2).

**Scheme 1:**
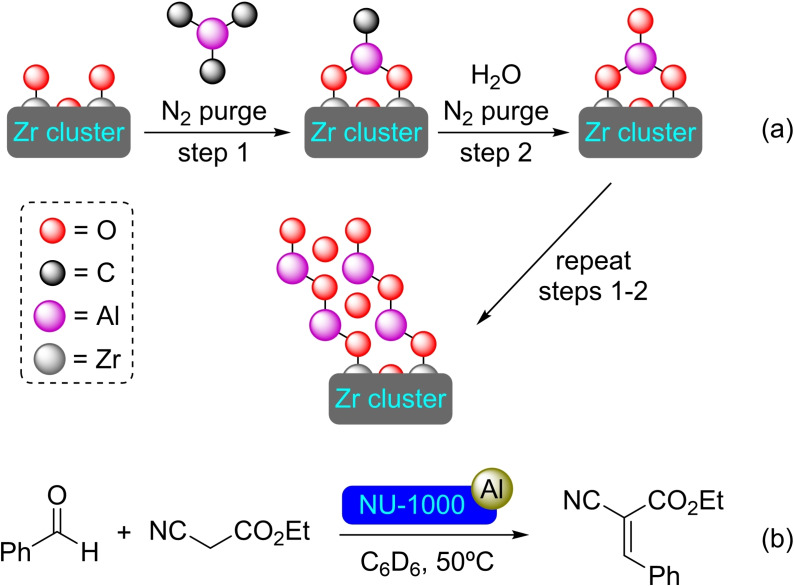
(a) Thin film ALD on a metal node (for simplicity hydrogen atoms are omitted), and (b) Knoevenagel condensation catalyzed by [Al]@NU‐1000.

**3.1.1. Aluminum modification**. NU‐1000 has been metalated with aluminum employing triethylaluminium (AlMe_3_) by ALD, attaching the aluminum to all the hydroxyl moieties present in the nodes with an Al/node ratio of 1.4 (Scheme 1). SIM with AlMe_3_ results in a material with similar characteristics. The highly Lewis‐acidic [Al]@NU‐1000 has proven to be active in the Knoevenagel condensation of ethyl cyanoacetate and benzaldehyde, being a prove of concept of its catalytic activity (Scheme 1).^[32]^ Similarly, the milder precursor dimethylaluminum isopropoxide (*i*‐PrOAlMe_2_) has also proven to be effective to prepare aluminum‐modified NU‐1000 by ALD while preserving the mesoporosity, crystallinity and surface area of the MOF. Aluminum oxide nanoclusters have been stabilized in the pores of the [Al]@NU‐1000, presenting spectroscopic similarities with γ‐Al_2_O_3_. Based on this resemblance, [Al]@NU‐1000 proved to catalyze ethanol dehydration with more selectivity towards ethene than γ‐Al_2_O_3_.^[33]^ Moreover, it has been determined *via* calculations that the amount of aluminum could be increased further by means of more prolonged ALD aided by partial node dehydration, up to the inclusion of eight Al atoms per node, increasing the potential catalytic ceiling of the material.^[34]^

**Scheme 2:**
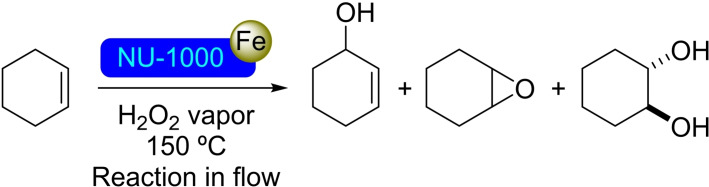
Vapor phase catalytic oxidation of cyclohexene with H_2_O_2_ catalyzed by [Fe]@NU‐1000.

**Figure 3:**
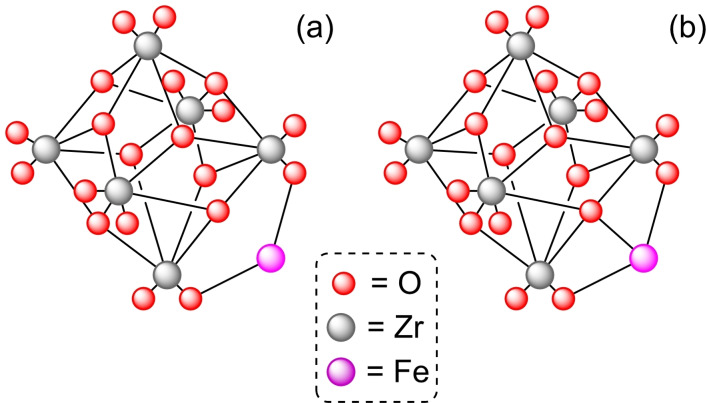
Models of node structure of iron insertion in the zirconium cluster: (a) Fe atom linked to two O atoms, and (b) Fe atom linked to three O atoms. For simplicity hydrogen atoms are omitted.

**3.1.2. Iron modification**. The metalation of NU‐1000 with iron has been successfully achieved by SIM of the MOF in solutions of Fe(NO_3_)_3_ or FeCl_2_, with iron loadings of 0.5 and 2.2 atoms per node, respectively. The crystalline structure of the MOF remained unaltered, the iron(III) single‐ion sites found in both cases indicate that the iron(II) precursor was oxidized to iron(III) during the metalation step.^[35]^ Both [Fe]@NU‐1000 catalysts were effective in the oxidation of cyclohexene with hydrogen peroxide in vapor‐phase, achieving a steady‐state stream of a mixture of products (i. e. the corresponding epoxide, diol, and allylic alcohol, Scheme 2). The insertion of iron(II) species in the nodes of NU‐1000 has also been achieved using a solution of potassium hexacyanoferrate(II) trihydrate. The loading of iron resulted to be 1.1 atoms per node, with retention of the crystallinity and mesoporosity.^[36]^ These iron species resulted in two crystallographically independent iron sites (Figure 3), being linked to two Zr‐OH groups (Fe1) or to two Zr‐OH groups and a third oxygen anion linked to three Zr atoms (Fe2).

**Scheme 3:**
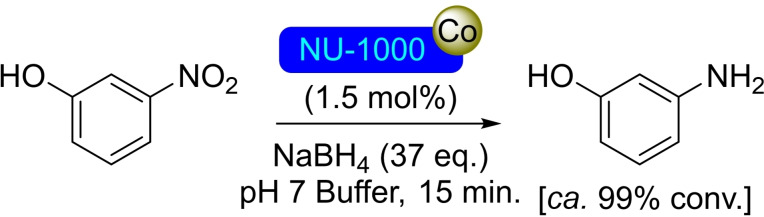
Catalytic reduction of nitrophenol with [CoS]@NU‐1000.

**Scheme 4:**
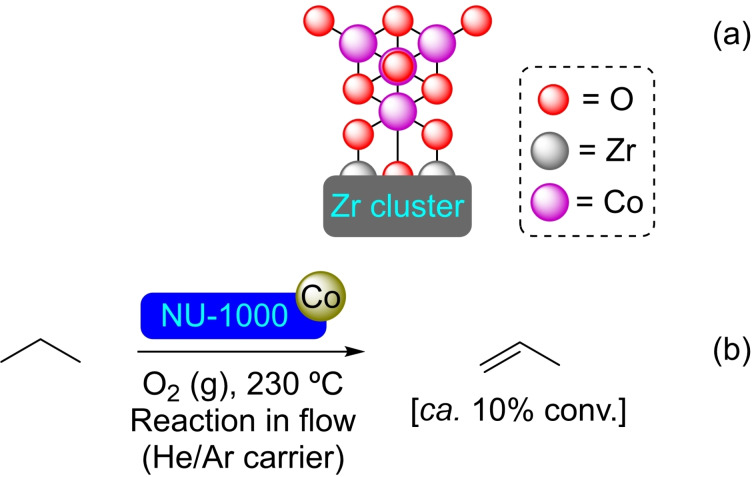
(a) A proposed spinel‐like structure of the catalytic cluster (for simplicity hydrogen atoms are omitted), and (b) catalytic oxidation of propane with [Co]@NU‐1000.

**3.1.3. Cobalt modification**. Cobalt sulfide deposition on NU‐1000 has been achieved by the ALD *via* slow diffusion of the precursors hydrogen sulfide (H_2_S) and bis(*N*,*N*’‐diisopropylacetamidinato)cobalt(II) [Co(MeC(NiPr)_2_)_2_] through the material, growing at the hydroxyl moieties in the nodes of the MOF and yielding the modified material [CoS]@NU‐1000. The ratio of cobalt and sulfur per node resulted in 1.2 and 1.1, respectively, although the former could be increased up to saturation with a value of 7.5 Co/node. The reduction of a nitro compound (i. e. 3‐nitrophenol) to the corresponding amino derivative employing sodium borohydride in the presence of 1.5 mol % of [CoS]@NU‐1000 was performed to prove its catalytic activity (Scheme 3). The conversion (in 15 min) to the reduced compound was quantitative, while plain cobalt sulfides produced incomplete conversion (30 %) and pristine NU‐1000 gave no reaction whatsoever.^[37]^ Similar [Co]@NU‐1000 materials prepared by ALD and SIM have been prepared, being postulated a spinel (Co_3_O_4_) structure for the active sites (Scheme 4). The catalytic activity of this material was proved in the dehydrogenation of propane to propene in the presence of O_2_ (Scheme 4), at considerably lower temperature (200 °C) than common supported catalysts (300–500 °C).^[38]^ The treatment of the NU‐1000 with naphthalenecarboxylic acid prior to cobalt addition, allowed the exclusive deposition of cobalt on the nodes, although did not result in any significant difference in terms of activity in the dehydrogenation reactions.^[39]^ Moreover, uniform NU‐1000 thin films grown on conducting glass (transparent fluoride‐doped tin oxide) electrodes have been modified by ALD, obtaining 3D arrays of metal‐ion heterogeneous catalysts. As proof of its application in electrochemical transformations, this functionalized material has been successfully used to promote the water oxidation reaction.^[40]^

**Scheme 5:**
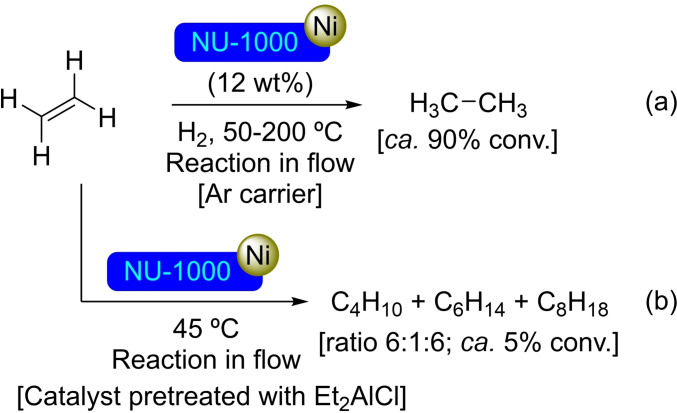
Catalytic reactions of [Ni]@NU‐1000: (a) Reduction of ethene, and (b) oligomerization of ethene.

**Figure 4:**
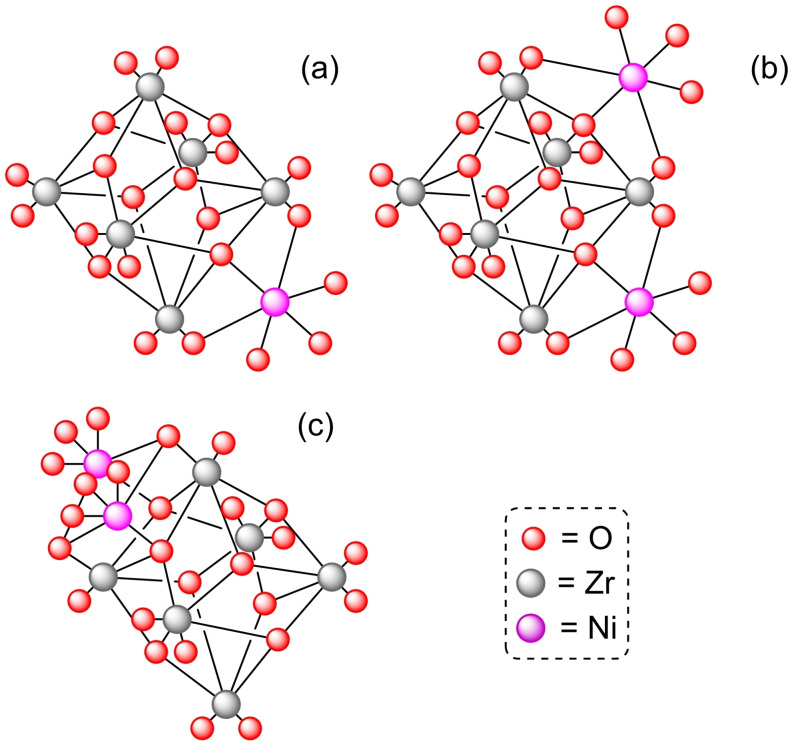
Calculated node structure for (a) one Ni atom per face with one Ni atom included, (b) one Ni atom per face with two Ni atoms included, and (c) two Ni atoms per face model. For simplicity hydrogen atoms are omitted.

**3.1.4. Nickel modification**. The compound bis(*N*,*N*’‐di‐*tert*‐butylacetamidinato)nickel(II) [Ni(MeC(N*t*‐Bu)_2_)_2_] has been employed as precursor for the deposition of nickel by ALD on NU‐1000.^[41]^ In combination with water up to four NiO(H) units have been formed per node. Additionally, solvothermal immersion of NU‐1000 in a solution of metal precursor, such as nickel(II) acetate^[42]^ or [Ni(MeC(N*t*‐Bu)_2_)_2_],^[43]^ has been reported as an efficient protocol for the deposition of nickel in the nodes. [Ni]@NU‐1000 has resulted to be a good catalyst in flow ethylene hydrogenation after being activated with hydrogen gas (3 % H_2_/Ar) at 200 °C^[41]^ or (4 % H_2_/Ar) at 220 °C (Scheme 5), remaining active during 2 weeks under flow reaction conditions.^[42]^ Mechanistic studies have suggested the presence of nickel hydride species, which are protected by the MOF structure. Even after deactivation by exposure to the atmosphere, they can easily be restored by treatment with a hydrogen/argon mixture. The amount of nickel in the nodes is related with the activity of the catalyst, showing markedly increased reactivity in the presence of Ni‐O−Ni sites. Thus, rates per nickel atom sharply increase when the loading reaches 2.1 nickel per node, continuing to increase up to 3.4 nickel atoms per node,^[43]^ with different possible coordination to the node (Figure 4). Based on that, [Ni]@NU‐1000 has been activated with diethylaluminum chloride (Et_2_AlCl) to form an effective catalyst for ethylene oligomerization forming C_4_, C_6_ and C_8_ products (Scheme 5).^[41]^ In a similar way, ALD employing [Ni(MeC(N*t*‐Bu)_2_)_2_] in combination with H_2_S has allowed for the preparation of a NU‐1000 derivative with nickel sulfide deposition, having an average of 4.2 nickel atoms and 4.7 sulfur atoms per node, obtaining a material suitable for the photocatalytic hydrogen evolution reaction.^[44]^

**Figure 5:**
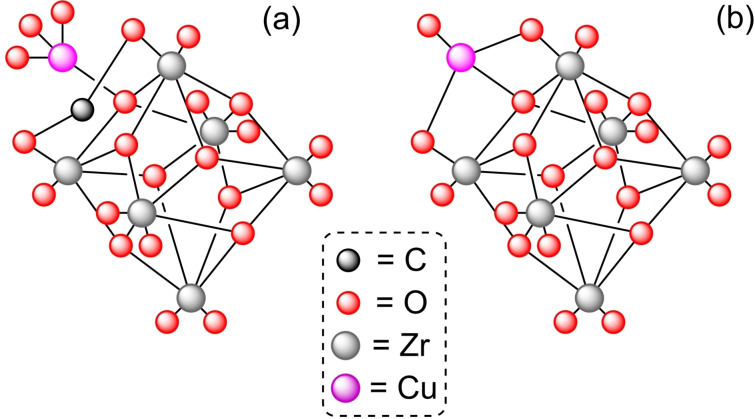
Models of node structure of copper insertion in the zirconium cluster: (a) with a formate unit, (b) without formate unit. For simplicity hydrogen atoms are omitted.

**3.1.5. Copper modification**. The modification of NU‐1000 with copper has been achieved using bis(dimethylamino‐2‐propoxy)copper(II) in combination with water by the ALD. [Cu]@NU‐1000 presents clusters of copper oxide (CuO) and copper hydroxide [Cu(OH)_2_], in a ratio 2.5/1, with a total incorporation of copper of about 4 to 4.5 atoms per node. Activation of [Cu]@NU‐1000 with an oxygen flow at high temperature (200 °C) enables it to mediate the oxidation of methane, observing the formation of methanol, dimethyl ether, and carbon dioxide.^[45]^ Moreover, the modified MOF [Cu]@NU‐1000 has been treated with a H_2_ flow during several cycles of heating (25 to 325 °C), bringing about the reduction of copper to Cu(0). The so‐formed copper atoms developed into larger copper nanoparticles (*ca*. 6 nm).^[46]^ Furthermore, it has been observed that Cu(II) ions in [Cu]@NU‐1000 are reduced to Cu(0) nanoparticles by heating under vacuum or in an inert atmosphere. HCl treatment in dimethylformamide (DMF) is a standard procedure after synthesis of a zirconium‐based MOF (i. e. NU‐1000) to remove synthesis modulators and unreacted species, but this action aids the partial installation of formate ligands onto the hydroxy moieties of the node (Figure 5). These formate units have been correlated with the spontaneous reduction of copper under thermal treatment.^[47]^

**Scheme 6:**
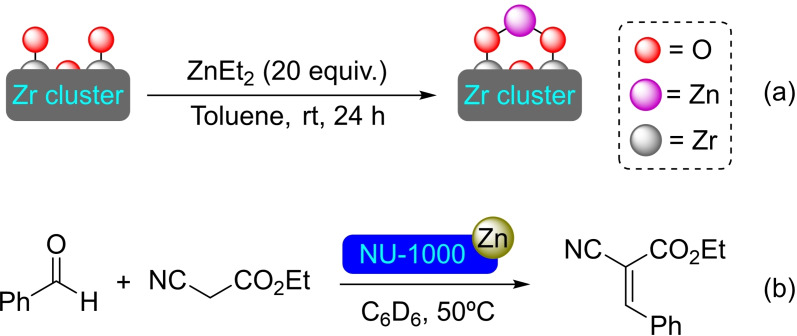
(a) [Zn]@NU‐1000 prepared by SIM (for simplicity hydrogen atoms are omitted), and (b) Knoevenagel condensation catalyzed by [Zn]@NU‐1000.

**3.1.6. Zinc modification**. NU‐1000 has been metalated with zinc by ALD using diethylzinc (ZnEt_2_) as precursor, resulting in nodes with a ratio of 0.5 Zn/Zr. Similar incorporation of zinc can be achieved by immersion of the material in a solution containing ZnEt_2_. Probably due to the size of the precursor, zinc only seems to interact with the hydroxyl moieties in the mesoporous channels. In addition, [Zn]@NU‐1000 was prepared by SIM, being successfully assayed in the Knoevenagel condensation between ethyl cyanoacetate and benzaldehyde (Scheme 6), where the unmodified NU‐1000 has no significant catalytic activity.^[32]^ Besides, [Zn]@NU‐1000 has resulted a versatile starting structure for the preparation of other metalated porous materials *via* the modification protocols described above. For instance, [Zn]@NU‐1000 has been immersed in a methanolic solution of copper(II) salts [i. e. CuCl_2_⋅2H_2_O, CuBr_2_, Cu(NO_3_)_2_⋅2.5H_2_O] resulting in all the cases in complete exchange of Zn(II) ions for Cu(II) ions. Similar procedures using NiCl_2_ has produced a 60 % of exchange (Ni for Zn), and CoCl_2_⋅6H_2_O has resulted in 90 % replacement of zinc by cobalt. These hybrid materials open the possibility for the development of synergistic multimetallic catalysts based on MOFs.^[48]^

**Scheme 7:**
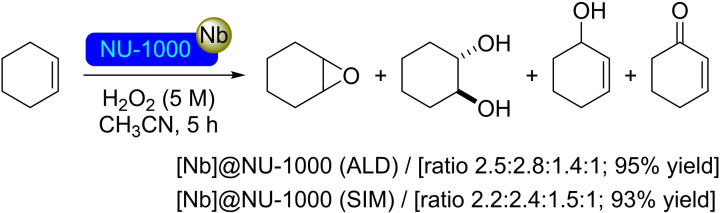
Catalytic oxidation of cyclohexene with [Nb]@NU‐1000 prepared by ALD or by SIM.

**3.1.7. Niobium modification**. The niobium modification of NU‐1000 has been achieved by using (*tert*‐butylimido)tris(diethylamido)niobium(V) (TBTDEN) as the metal precursor by both ALD and SIM. The atomic layer deposition of niobium has been performed at 110 °C, being subsequently treated with H_2_O at room temperature to form isolated niobium oxide centers on the nodes. Solution deposition was performed by dissolving the metal precursor in heptane and then exposing the modified material to an air atmosphere during washing. In both cases, the niobium(V) content is 4 atoms per node, with further increases in the metal loading in NU‐1000 leading to a degradation of the structure. Regardless of its preparation method, [Nb]@NU‐1000 has shown high activity in the oxidation of cyclohexane using hydrogen peroxide (Scheme 7). A mixture of different products (such as alkene oxide, 1,2‐diol, allyl alcohol, and enone) was obtained, with better activity being observed when the particle size is reduced, suggesting that the process may be taking place in the surface of the material, rather than inside the pores.^[49]^

**Scheme 8:**
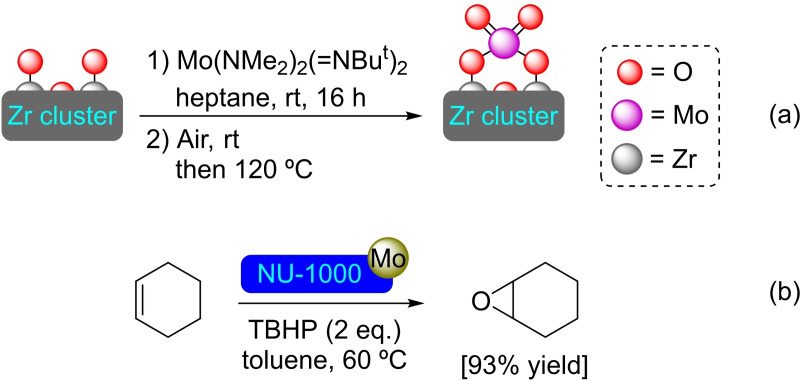
(a) [Mo]@NU‐1000 prepared by SIM (for simplicity hydrogen atoms are omitted), and (b) catalytic epoxidation of cyclohexene with [Mo]@NU‐1000.

**3.1.8. Molybdenum modification**. The solvothermal metalation of NU‐1000 with bis(*tert*‐butylimido)bis(dimethylamino)molybdenum(VI) (TBTDEM) followed by exposure to oxygen has been employed to prepare a molybdenum(VI) oxide deposited MOF (Scheme 8). This [Mo]@NU‐1000 presents Mo monomeric species or clusters of a few Mo atoms, with a ratio of 2.8 Mo atoms per node. The material has resulted active in the epoxidation of alkenes, where NU‐1000 shows no activity. High conversions have been observed for cyclic olefins, such as cyclohexene (93 %) and cyclooctene (>99 %), with moderate results for acyclic hex‐1‐ene (55 %). In all cases, the alkene was transformed in a mixture of the corresponding alkene oxide and the 1,2‐diol (by a ring opening reaction with residual H_2_O), with a selectivity greater than 99 % for the mixture of both products (Scheme 8).^[50]^ Likewise, a sulfur‐containing molybdenum deposited NU‐1000 has been prepared by exposure to H_2_S after the metalation instead of oxygen. The material prepared in this case (i. e. [MoS]@NU‐1000) has mononuclear MoS_x_ or small clusters of few Mo atoms present, with a 2.6 ratio of Mo per node. The material is electrocatalytically active, allowing the formation of hydrogen gas from acidified water. The initially low electrocatalytic activity was enhanced using redox mediators in solution.^[51]^ MoS_2_ has also been introduced by ALD, using molybdenum hexacarbonyl and hydrogen sulfide in successive cycles. The modified material has high activity and high stability in the direct desulfurization of dibenzothiophene, offering itself as a new class of hydrodesulfurization catalyst.^[52]^

**Scheme 9:**
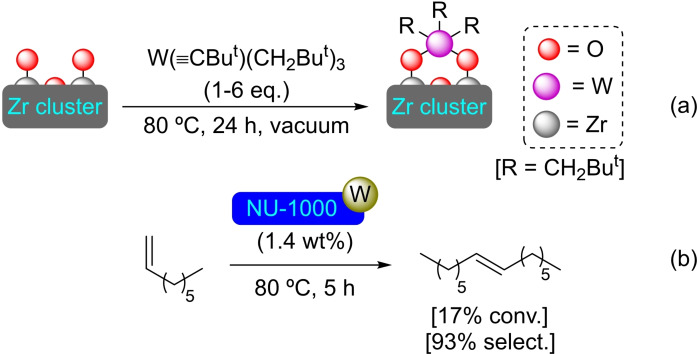
(a) [W]@NU‐1000 preparation by SIM (for simplicity hydrogen atoms are omitted), and (b) selective metathesis of 1‐octene catalyzed by [W]@NU‐1000.

**3.1.9. Tungsten modification**. Heat treatment (80 °C) under vacuum of a thorough mix of NU‐1000 with an excess of W(≡C*t‐*Bu)(CH_2_ *t‐*Bu)_3_ has resulted in the uniform incorporation of tungsten species, with the loss of a molecule of neopentane per complex affixed (Scheme 9). In the [W]@NU‐1000 material, the W per node ratio is between 0.5 and 1, with tungsten having been incorporated as an alkyl‐W=O complex. The material is active in the metathesis of alkenes. Its activity has been tested in the metathesis of propylene both in a batch reactor and in a flow reactor, revealing the generation of active carbene sites of W. The study of the conversion of 1‐octene to 7‐tetradecene has exposed that the catalyst needs an activation period (2–3 h) to give a rapid formation of 7‐tetradecene achieving maximum of selectivity after 5 h (Scheme 9).^[53]^

**Scheme 10:**
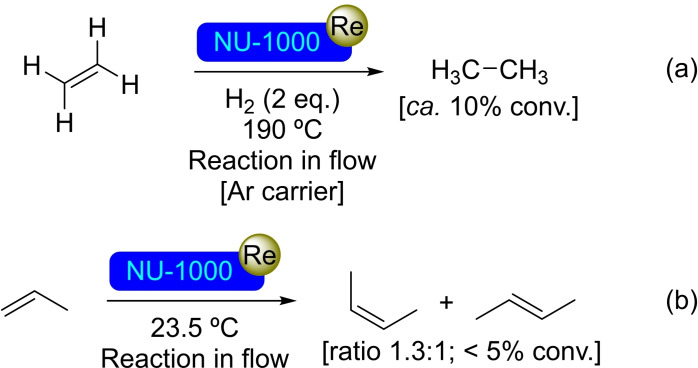
Catalytic reactions of [Re]@NU‐1000: (a) Reduction of ethene, and (b) metathesis of propene.

**3.1.10. Rhenium modification**. Methyltrioxorhenium (MeReO_3_) has been used as precursor for ALD of rhenium oxide in NU‐1000.^[54]^ The metalated MOF, [Re]@NU‐1000 has proved to be active for ethene hydrogenation in a gas‐phase flow reactor (Scheme 10). More recently, the possibility of preparing the [Re]@NU‐1000 by ALD at lower temperature has been verified, obtaining a MOF with a rhenium per node ratio of 1 : 1. The material resulted an active catalyst in olefin metathesis, with the Lewis acidity of the nodes having a crucial synergistic effect in the catalytic activity of the material.^[55]^ The metathesis of propene resulted in the formation of a mixture of isomers of but‐2‐ene with preference for the less thermodynamically stable one (Scheme 10).

**Scheme 11:**
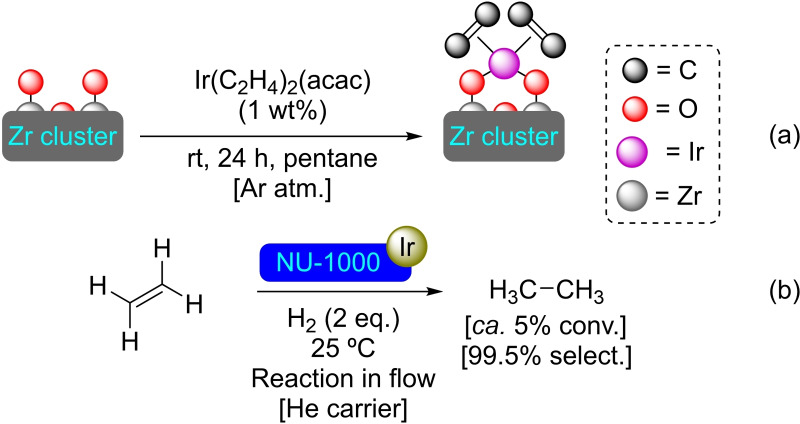
(a) [Ir]@NU‐1000 preparation with Ir(C_2_H_4_)_2_(acac) [acac=acetylacetonate] (for simplicity hydrogen atoms are omitted), and (b) catalytic hydrogenation of ethene with [Ir]@NU‐1000.

**3.1.11. Iridium modification**. Iridium complexes [such as Ir(CO)_2_ and Ir(C_2_H_4_)_2_] have been supported on the nodes of NU‐1000 by SIM. The iridium loading on the MOF was established to be 10 wt % for the Ir(CO)_2_ and 1 wt % for the Ir(C_2_H_4_)_2_, with the iridium bonding to the nodes in the MOF structure (Scheme 11). The supported Ir(C_2_H_4_)_2_ has shown catalytic activity in the hydrogenation of ethylene in a flow reactor with a selectivity of 99.5 % to ethane (Scheme 11). This catalyst was observed to be comparable in terms of active sites to the fresh catalyst after 1 h of continuous hydrogenation reaction, proving its high stability under the reaction conditions.^[56]^

**3.1.12. Platinum modification**. The ALD of trimethyl(methylcyclopentadienyl)platinum(IV) to NU‐1000 has been employed to prepare platinum modified MOFs. The preparation of [Pt]@NU‐1000 has been performed at 115 °C and 160 °C observing a significant difference in the loading of metal, being 0.15 Pt and 2.5 Pt per node, respectively. Pt atoms are present as single‐sites or few atom clusters. The resulting material, [Pt]@NU‐1000 is active in the hydrogenation of ethylene.^[57]^

**3.1.13. Uranium modification**. The uranyl ion (UO_2_)^2+^ is a stable form of U(VI) in which the metal is bonded to two oxygen atoms axially. This cation can be deposited on several materials, obtaining active catalysts for oxidation processes. Solvothermal deposition of this ion on NU‐1000 has been achieved using uranyl acetate, reaching a loading of 1.3 (UO_2_)^2+^ ions per MOF node. The [(UO_2_)^2+^]@NU‐1000 material has been tested in the photocatalyzed oxidation of 4‐methoxybenzyl alcohol showing reduced activity compared to plain NU‐1000 or uranyl acetate. This could be attributed to interference between both photoactive species, rather than the desirable synergy.^[58]^

**Figure 6:**
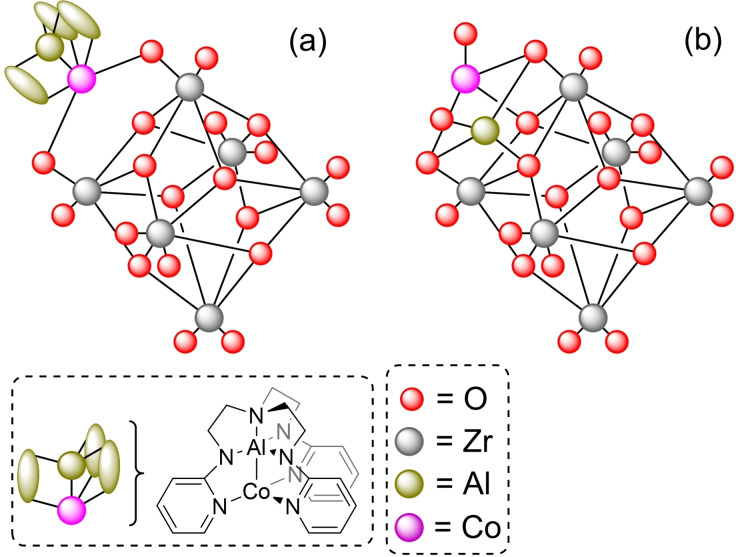
Models of node structure of bimetallic species in the zirconium cluster: (a) (py_3_tren)AlCo complex, (b) AlCo oxide cluster. For simplicity hydrogen atoms are omitted.

**3.1.14. Bimetallic modification**. As mentioned before, the deposition of bimetallic species can have a synergistic effect that enhances the catalytic properties of the material beyond the mere sum of the components. In this sense, a bimetallic complex of aluminum and cobalt, with the heptadentate ligand *N*,*N*,*N*‐tris(2‐(2‐pyridylamino)ethyl)amine (py_3_tren), has been used to deposit aluminum‐cobalt species on NU‐1000 by SIM. The (py_3_tren)AlCo metal complex is attached to the hydroxyl residues of the nodes forming Al‐Co−O linkages, with a 1 : 1 ratio of AlCo per node (Figure 6). Heat treatment of the material (300 °C) in air atmosphere causes the transformation of the deposited complexes into clusters of cobalt‐aluminum hydroxide [CoAl(OH)_2_] (Figure 6). Both modified materials can catalyze the oxidation of benzyl alcohol to benzaldehyde with *tert*‐butyl hydroperoxide, with cobalt seemingly having a pivotal effect on the catalyst's activity.^[59]^ In this sense, NU‐1000 has been modified with different metals (i. e. nickel, zinc, aluminum, titanium, and molybdenum) by SIM, and then modified further with cobalt(II) ions by ALD. The generated [Co][Metal]@NU‐1000 have a ratio of 3–4 metal ions (i. e. Ni, Zn, Al, Ti, or Mo) deposited onto the zirconium node and 6–8 Co atoms deposited on top of the first metal used. This way, the metal modulates the catalytic activity of cobalt in the oxidation of propane to propene, as the [Metal]@NU‐1000 materials are not active in this transformation, with the observable activity trend [Ni(II) > Zn(II) > Al(III) > Ti(IV) > Mo(VI)] being inverse to the Lewis acidity of the ions.^[60]^ As further proof of the potential of bimetallic modifications, it has also been described that the combination of cobalt and zinc in NU‐1000 results in a significant difference in chemoselectivity during the reduction of propyne. Indeed, the [Zn][Co]@NU‐1000 material, prepared by consecutive ALD of cobalt and zinc, shows decreased activity towards propene formation compared to other metal and bimetallic catalysts deposited on NU‐1000, favoring isomerization to propadiene instead. Moreover, this [Zn][Co]@NU‐1000 also reduced oligomerization and coking, which are undesirable processes in this type of transformation.^[61]^

**Scheme 12:**
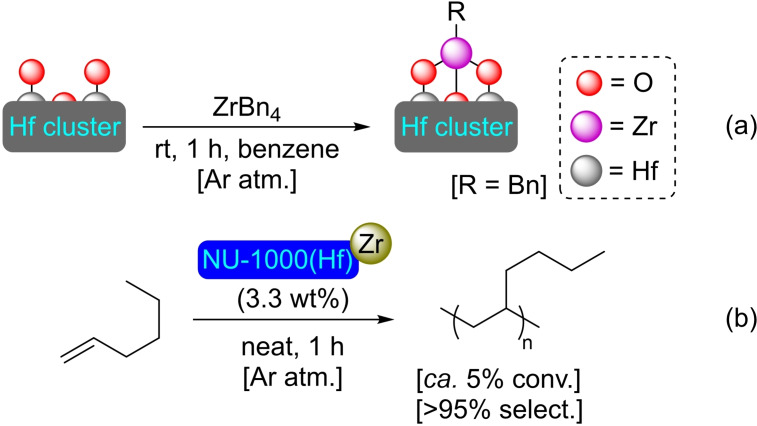
(a) Preparation of [Zr]@NU‐1000(Hf) by SIM (for simplicity hydrogen atoms are omitted), and (b) catalytic polymerization of 1‐hexene.

**3.1.15. Zirconium modification of NU‐1000 based on hafnium**. Hafnium is similar in terms of behavior and coordination geometry to zirconium. This allows for the preparation of MOFs isostructural to their zirconium counterparts, such as NU‐1000(Hf).^[62]^ This hafnium‐based MOF has been treated with tetrabenzylzirconium (ZrBn_4_) resulting in the incorporation of 2.4 Zr atoms per node (Scheme 12). The strongly Lewis‐acidic benzyl‐Zr species provide [Zr]@NU‐1000(Hf) with catalytic activity in olefin polymerization reactions (Scheme 12), selectively affording polyethylene and isotactic‐poly(1‐hexene) from ethylene and 1‐hexene, respectively.^[63]^

### 3.2 NU‐1200

The combination of the linker 4,4′,4′′‐(2,4,6‐trimethylbenzene‐1,3,5‐triyl)tribenzoic acid with zirconium (or hafnium) results in NU‐1200, a porous material with sodalite‐like cage topology. The presence of the methyl groups in the linker increases the steric demand on the benzenoid core ring forcing the benzoic acid groups to be perpendicular to the core ring. The material presents ‐OH and H_2_O groups, attached to the unsaturated Zr_6_ nodes pointing to the mesoporous channel, offering a clear point for functionalization. Indeed, these positions have been used to bind titanium(IV) cations by SIM using titanium tetraisopropoxide, although the material has not been tested in catalysis.^[64]^ Later, the deposition of other metals (i. e., nickel and molybdenum) has resulted in materials that have been tested for their catalytic activity.

**Scheme 13:**
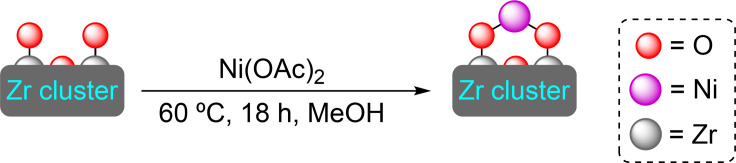
(a) [Ni]@NU‐1200 prepared by SIM with Ni(OAc)_2_ (for simplicity hydrogen atoms are omitted).


**3.2.1. Nickel modification**. Nickel has been deposited in both zirconium and hafnium NU‐1200 by SIM with a Ni(II) salt. As in the case of NU‐1000, nickel is attached to the nodes through oxo bridges, with a ratio of 1 nickel atom per node (Scheme 13). This binding form facilitates the formation of nickel hydride species by exposure to hydrogen gas, with the channels protecting the active species from the environment. Indeed, the [Ni]@NU‐1200 materials proved very effective for the hydrogenation of ethylene, with similar activity to that of [Ni]@NU‐1000 (Scheme 5). In all cases, the activity is significantly higher than on materials with monodentate nickel binding.^[42]^

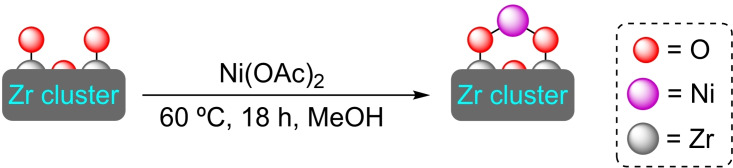


**Scheme 14:**
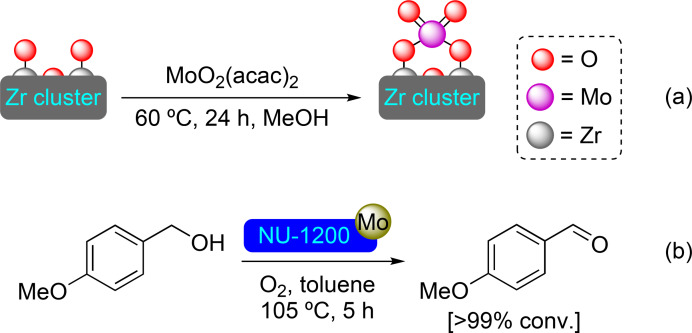
(a) [Mo]@NU‐1200 prepared by SIM with MoO_2_(acac) [acac=acetylacetonate] (for simplicity hydrogen atoms are omitted), and (b) catalytic oxidation of 4‐methoxybenzyl alcohol with [Mo]@NU‐1200.

**3.2.2. Molybdenum modification**. Molybdenum has been grafted onto NU‐1200 by SIM using (acetylacetonate)dioxomolybdenum(VI) as a precursor. [Mo]@NU‐1200 exhibits a ratio of 1.2 Mo atoms per node (Scheme 14). Analysis of the material reveals two distinct coordination modes for Mo atoms at the node, although with no change in oxidation state. Part of the molybdenum atoms are coordinated in an octahedral geometry with a terminal oxygen atom of one node and two –OH groups with the oxygen oriented to the mesopore. The rest present a tetrahedral geometry with four oxygen atoms, two being adjacent terminal oxygens at the node and the other two oriented to the mesopore, like that observed in molybdenum deposition on zeolites. [Mo]@NU‐1200 has proved its catalytic activity in the oxidation reaction of 4‐methoxybenzyl alcohol under an oxygen (O_2_) atmosphere, with complete conversion in just 5 h (Scheme 14). Interestingly, the two binding motifs exhibit different activities, with octahedral centers being more efficient than their tetrahedral counterparts.^[65]^

### 3.3 UiO‐66

**Figure 7:**
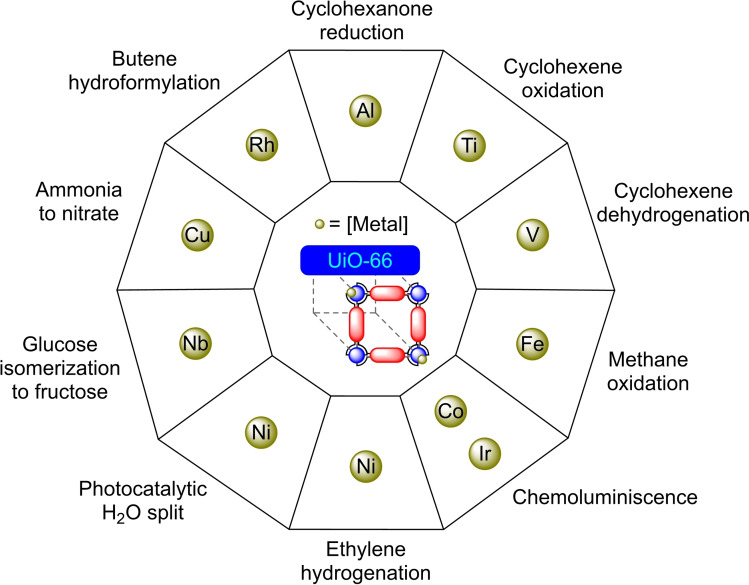
Post‐synthetic modifications of UiO‐66 (including UiO‐66‐NH_2_) with metals and their applications.

UiO‐66 was first reported in 2008 by Lillerud's group. UiO‐66 is composed of zirconium oxide nodes connected by terephthalic acid ligands (BDC). Due to its easy synthesis, ready availability of the starting materials and relatively high stability, UiO‐66 is one of the most widespread and well‐studied MOFs.^[66]^ The PSM with metals has provided a variety of materials with different catalytic applications (Figure 7).

**Scheme 15:**
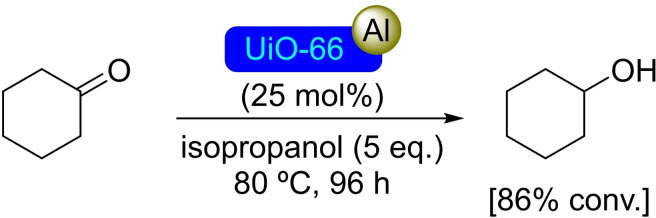
Catalytic Meerwein‐Ponndorf‐Verley oxidation with [Al]@UiO‐66.

**3.3.1. Aluminum modification**. UiO‐66 has been modified by treatment with trimethylaluminum to load aluminum centers and then transform them into the corresponding alkoxides by treatment with isopropanol. This type of post‐synthetic modification is straightforward due to the acidity of the hydroxyl sites of this type of MOF. The catalytic activity of [Al]@UiO‐66 has been tested in the reduction of cyclohexanone in a Meerwein‐Ponndorf‐Verley process, producing cyclohexanol with good conversion at room temperature after 96 h of reaction (Scheme 15). The unmodified material is completely inactive at room temperature, only showing some above 80 °C due to the presence of defects with Lewis acidity in the structure.^[67]^ The use of 2‐aminoterephthalic acid as a ligand results in an analogous material with free amino groups on the linkers (UiO‐66‐NH_2_). Interestingly, the deposition of Al seems to occur selectively at the nodes when using trimethylaluminum as precursor by ALD, with amino groups remaining untouched.^[68]^

**Scheme 16:**
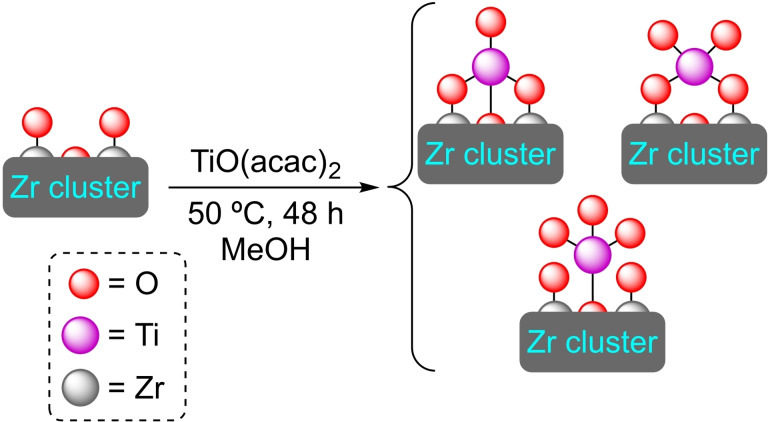
[Ti]@UiO‐66 prepared by SIM with TiO(acac)_2_ [acac=acetylacetonate], showing possible coordination modes of titanium species. For simplicity hydrogen atoms are omitted.

**3.3.2. Titanium modification**. [Ti]@UiO‐66 has been prepared by treatment of the pristine MOF with a methanolic solution of TiO(acac)_2_. Titanium is attached to the MOF nodes by hydroxyl groups with a ratio of 0.24 titanium atoms per node (Scheme 16). This [Ti]@UiO‐66 has been shown to be catalytically active in the oxidation of cyclohexene with hydrogen peroxide, forming mainly the corresponding allylic oxidation products (cyclohex‐2‐en‐1‐ol and cyclohex‐2‐enone). The heterogeneous catalyst can be recycled, but the activity is gradually lost (75 % activity in the third cycle) due to titanium leaching. In this work, all forms of Ti modification were assayed and compared. In addition to the first material prepared, various linkers (up to 46 %) were exchanged for 2,3‐dihydroxyterephthalate to subsequently bind titanium as a catechol complex. Titanium atoms have also been inserted into the node by PSE. Both modified materials are less active than the one prepared by titanium bonding to the node, showcasing the advantages of direct node modification.^[69]^ Titanium can also be inserted by ALD, as UiO‐66‐NH_2_ has also been successively treated with titanium chloride and water to obtain a titanium modified material,^[68]^ although it has not been tested as catalyst.

**Scheme 17:**
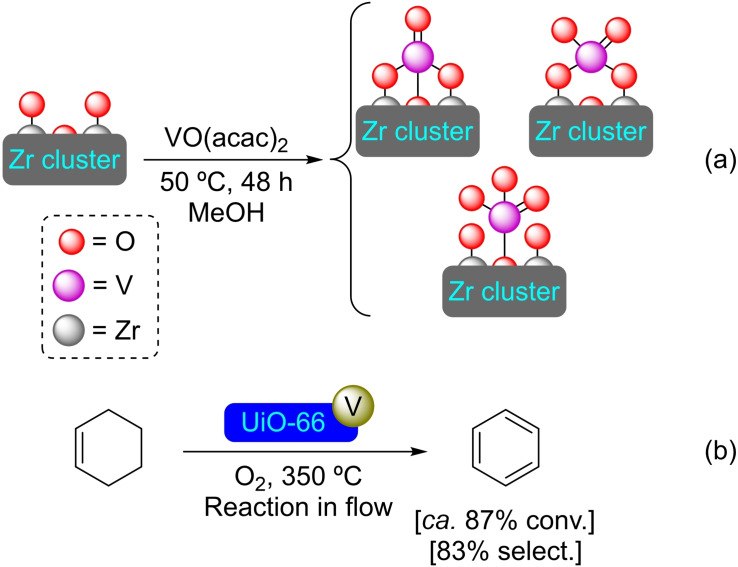
(a) Possible coordination modes of vanadium in [V]@UiO‐66 prepared by SIM with VO(acac)_2_ [acac=acetylacetonate] (for simplicity hydrogen atoms are omitted), and (b) selective oxidation of cyclohexene with [V]@UiO‐66.

**3.3.3. Vanadium modification**. Metalation of the UiO‐66 nodes has been achieved by SIM with a methanol solution of VO(acac)_2_, resulting in the incorporation of roughly 0.40 V atoms per node (Scheme 17a). This material, [V]@UiO‐66, can effectively catalyze the dehydrogenation of cyclohexene to give benzene, with higher conversions at higher temperatures (Scheme 17b). The activity and selectivity of this heterogeneous catalyst are very similar to those of vanadium oxides supported on alumina, suggesting a similar type of molecular arrangement.^[70]^

**Scheme 18:**
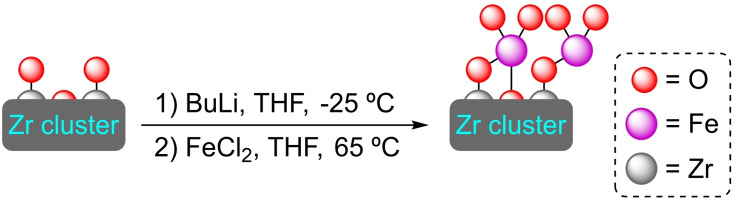
Models of coordination and preparation of [Fe]@UiO‐66. For simplicity hydrogen atoms are omitted.

**3.3.4. Iron modification**. An example of the metalation of UiO‐66 with iron has been recently described, following a simple deprotonation‐SIM procedure (Scheme 18). In that work, a series of [Fe]@UiO‐66 materials were prepared using either acetic acid or trifluoro acetic acid as modulators. This has a dramatic effect on the catalytic activity of the materials as [Fe]@UiO‐66_[TFA]_ shows up to 8‐fold increased performance on direct methane oxidation with aqueous H_2_O_2_ compared to is acetate‐bearing homologue. This is theorized to occur due to the highly electron‐deficient trifluoroacetate units grafted to the node stabilizing high‐oxidation state Fe species.^[71]^

**3.3.5. Cobalt modification**. As mentioned in the introduction section, highly defective MOF structures can be readily obtained by modulated syntheses. Obtained as such, a highly defective UiO‐66‐NH_2_ was treated with a solution of cobalt(II) chloride hexahydrate, resulting in a material loaded with highly active single Co atom sites. The so‐prepared [Co]@UiO‐66‐NH_2_ presented Fenton‐like activity, which boosted the signal emission of the redox‐based chemiluminescent reaction of the luminol‐H_2_O_2_ system by generating massive amounts of reactive oxygen species.^[72]^

**Scheme 19:**
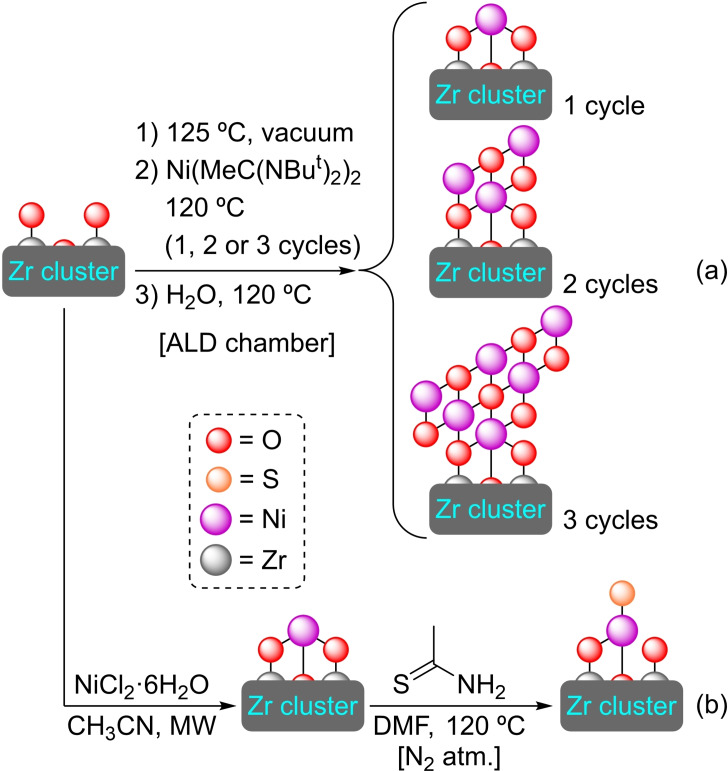
(a) Schematic representations of nickel(II) sites in [Ni]@UiO‐66 prepared by ALD, and (b) proposed structure of sulfur‐modified nickel(II) site in [Ni]@UiO‐66. For simplicity aqua ligands and hydrogen atoms are omitted.

**3.3.6. Nickel modification**. The deposition process of nickel(II) oxides has been achieved in UiO‐66 by ALD, with the average amount of nickel atoms per node varying according to the number of ALD cycles carried out (1.1, 1.3, or 1.6, for 1, 2 or 3 cycles, respectively). Interestingly, the deposition in the second and third cycles mostly occurs at the sites where nickel oxide is already present, increasing the size of the particles (Scheme 19). The modified materials were tested in the ethylene hydrogenation reaction, observing activities like those of other supported nickel catalysts. The catalysts are stable in the atmosphere of the hydrogenation reaction but are deactivated upon contact with air.^[73]^ A different approach for the modification of UiO MOFs with nickel is the microwave‐assisted SIM of UiO‐66‐NH_2_ with nickel(II) chloride hexahydrate which interestingly allows for the obtention of single dispersed nickel sites in the material. The environment of those nickel single‐sites can be further controlled by post‐synthetic treatment with thioacetamide or ammonia, resulting in the formation of highly active nickel(I) species (Scheme 19). For instance, materials involving sulfur‐coordinated nickel(I) sites exhibited excellent photocatalytic hydrogen production by water splitting under visible light.^[74]^

**Scheme 20:**
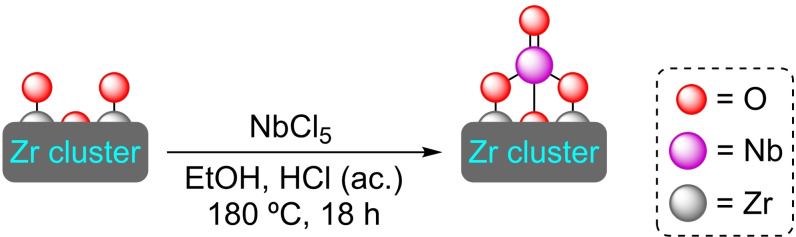
Model of coordination and preparation of [Nb]@UiO‐66 by SIM. For simplicity hydrogen atoms are omitted.

**3.3.7. Niobium modification**. Niobium species can be stabilized at Zr‐oxo cluster moieties and has thus been described in UiO‐66 by SIM with niobium(V) chloride (Scheme 20). The material tolerates the inclusion of different amounts of Nb (in the 3–7 wt % range) while maintaining the crystallinity of the structure. The incorporation of niobium in the UiO‐66 framework resulted in increased Lewis acidity and more acid sites in the material, improving twofold the conversion and selectivity of the isomerization reaction of glucose to fructose compared to unmodified UiO‐66. [Nb]@UiO66 with 5 % of Nb was found to have the optimum niobium‐zirconium ratio of active sites, affording the best fructose yield.^[75]^

**Scheme 21:**
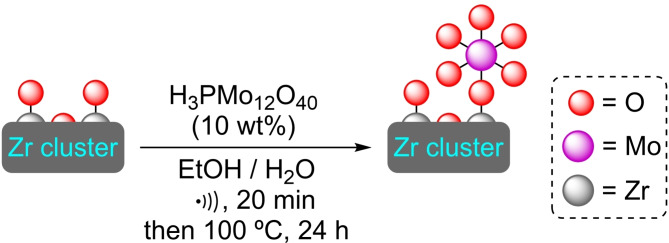
Model of coordination and preparation of [Mo]@UiO‐66 by SIM. For simplicity hydrogen atoms are omitted.

**3.3.8. Molybdenum modification**. Keggin‐type polyoxometalates (POM) are of great interest in catalysis due to their high activities arising from the presence of single atoms. However, they face issues related to low specific surface areas, solubility, and stability. In this context, phosphomolybdic acid (H_3_PMo_12_O_40_, PMA) was loaded in hierarchically porous UiO‐66 by ultrasound‐assisted SIM to afford a series of [Mo]@UiO‐66 materials, with most PMA units located at defects in the structure (Scheme 21). These materials were assayed for the oxidative desulfurization reaction in a series of fuel‐like solvents, achieving excellent results and stability up to 5 catalytic cycles. The optimum amount of PMA was found to be 10 % wt., with higher amounts hampering reactivity through aggregation and clogging of the pores of the material. The Lewis‐acidic Zr sites and the highly electron‐rich Mo atoms have been postulated to be key for the performance of the system.^[76]^

**Scheme 22:**
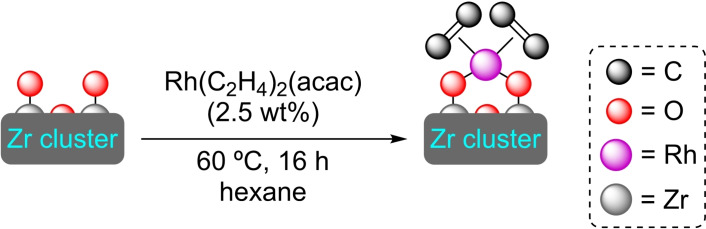
Model of coordination and preparation of [Rh]@UiO‐66 by SIM. For simplicity hydrogen atoms are omitted.

**3.3.9. Rhodium modification**. Rhodium can be inserted at the nodes of UiO‐66 by SIM with Rh(acac)(C_2_H_4_)_2_ (Scheme 22). This way, [Rh]@UiO66 containing ca. 1 wt % of active Rh was tested in the hydroformylation of butene, giving high conversion (95 %) although with low selectivity (25 %) to the expected pentanal.^[77]^

**Scheme 23:**
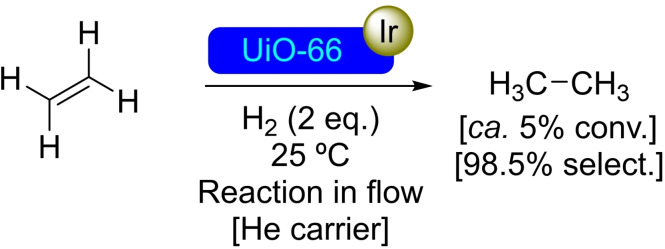
Catalytic hydrogenation of ethene with [Ir]@UiO‐66.

**3.3.10. Iridium modification**. SIM of UiO‐66 with a solution of Ir(CO)_2_ and Ir(C_2_H_4_)_2_ has allowed the incorporation of iridium species to the nodes of the MOF. In contrast with other supports (such as NU‐1000), the iridium seems to bond in different positions, mostly at defects on the nodes. Regardless, the [Ir]@UiO‐66 materials were found to promote the hydrogenation of ethylene to similar results than [Ir]@NU‐1000. The supported Ir(C_2_H_4_)_2_ was the most effective, showing 98.5 % selectivity towards the formation of ethane (Scheme 23).^[56]^ Grafting iridium complexes in MOFs is interesting, as the support node effectively acts as ligand for the iridium complex. Thus, its activity can be tuned through modification of the node itself (e. g. by modulation), in a very similar fashion to that described above for [Fe]@UiO‐66_[TFA]_.^[78]^

**Scheme 24:**
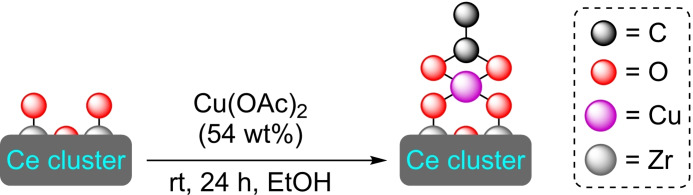
[Cu]@UiO‐66(Ce) preparation with Cu(OAc)_2_, and proposed structure model for single‐atom copper anchored to cerium cluster. For simplicity hydrogen atoms are omitted.

**3.3.11. Copper modification of UiO‐66 based on cerium**. Similarly to hafnium, cerium can be used to prepare UiO‐66 type frameworks with [Ce_6_O_4_(OH)_4_] clusters.^[79]^ Like his zirconium and hafnium counterparts, UiO‐66(Ce) presents high chemical and thermal stability, being a reasonable candidate for metal post‐modification. Thus, the modification by SIM of UiO‐66(Ce) using copper(II) acetate has been described (Scheme 24). The resulting material, [Cu]@UiO‐66(Ce) bears node‐grafted copper nanoclusters with controlled aggregation, avoiding higher sized particles. The functionalized material exhibits high selectivity (93 %) in the ammonia‐to‐nitrate conversion, a promising result for MOF‐based electrocatalysts with application to the ammonia synthesis.^[80]^

### 3.4 UiO‐67 Modified with Aluminum

**Scheme 25:**
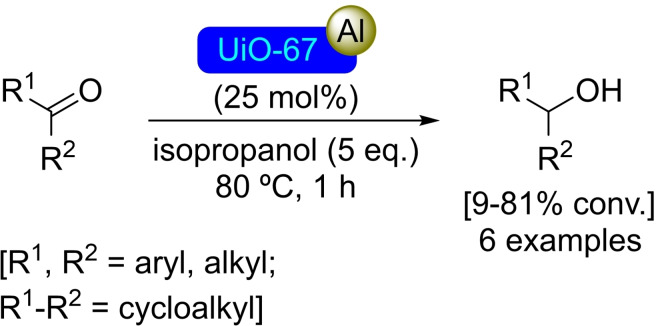
Catalytic Meerwein‐Ponndorf‐Verley oxidation with [Al]@UiO‐67.

The UiO‐67 MOF is isostructural to UiO‐66, consisting of Zr_6_O_4_(OH)_4_ nodes linked by biphenyl‐4,4′‐dicarboxylate (bdpc) units forming an octahedral cage, which is in turn surrounded by eight tetrahedral cages and another eight octahedral cages. The linker is longer in length than in UiO‐66, which increases the surface area of the material and average pore size while maintaining reasonable thermal and mechanical stability.^[81]^ As described for UiO‐66, the material UiO‐67 has been modified with aluminum isopropoxide centers by successive treatment with trimethylaluminum in solution and isopropanol. The [Al]@UiO‐67 material is active for the reduction of aldehydes and ketones by a Meerwein‐Ponndorf‐Verley reaction, noting low activity for ketones (i. e., acetophenone, benzophenone), while the case of aldehydes, it has been observed that larger substrates (i. e. dodecanal) were more complicated to reduce due to steric interactions, opening a possibility for chemo‐ and size‐selective catalysis (Scheme 25). The catalyst [Al]@UiO‐67 could be recycled and reused without loss of activity.^[67]^

### 3.5 UiO‐68

UiO‐68 is based on the same type of zirconium cluster as UiO‐66 and UiO‐67 materials but with longer para‐terphenyl‐4,4′′‐dicarboxylate (tpdc) linker, resulting in even bigger pore channels and more extensive specific surface area. The stability of this material is still high due to the highly compatible nodes and linkers, although it is substantially lower than that of UiO‐66 and UiO‐67 materials due to the excessive length of the latter.^[8]^

**Scheme 26:**
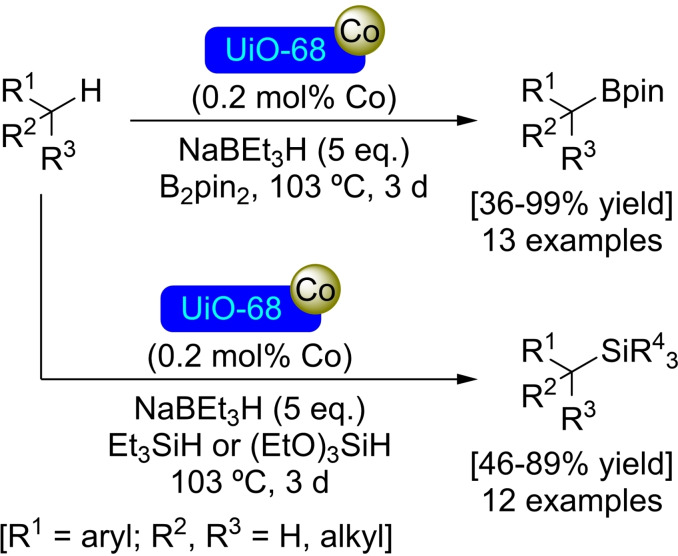
Catalytic benzylic borylation and silylation with [Co]@UiO‐68.

**Scheme 27:**
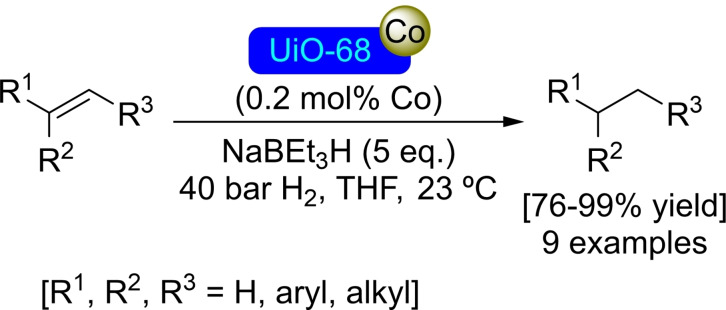
Catalytic hydrogenation of olefins [Co]@UiO‐68.

**3.5.1. Cobalt modification**. Metalation at the nodes of UiO‐68 is possible in the same way as in its analogues. In this sense, treatment of UiO‐68 with butyllithium followed by reaction with cobalt(II) chloride allows for the preparation of [Co]@UiO‐68, with a ratio of 4 cobalt atoms per node, while maintaining the structure of the material. Further treatment of [Co]@UiO‐68 with NaEt_3_BH results in the formation of an active cobalt hydride catalyst, which enables the borylation and silylation of benzylic C−H bonds employing bis(pinacolato)diboron [B_2_(pin)_2_] and triethylsilane or triethoxysilane[Et_3_SiH, (EtO)_3_SiH], respectively (Scheme 26). Similarly, this activated catalyst and in the presence of H_2_ (40 bar) allowed the hydrogenation of mono‐, di‐ and trisubstituted olefins (Scheme 27). In addition, [Co−H]@UiO‐68 is also active in the hydroboration of alkenes and carbonyl compounds with pinacolborane [HBpin].^[82]^

**Scheme 28:**
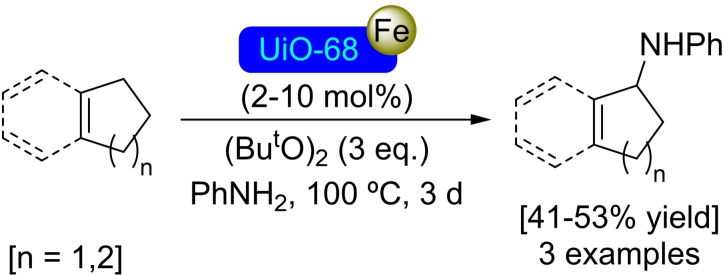
Catalytic amination of C−H bonds with [Fe]@UiO‐68.

**3.5.2. Iron modification**. Following the same procedure described above for the cobalt modification, the use of iron(II) bromide has allowed the preparation of [Fe]@UiO‐68, with 4 iron atoms per node. This material has been used as a catalyst in the amination of C−H bonds (benzylic or allylic) using aniline as a nitrogen source (Scheme 28).^[82]^

### 3.6 UiO‐69 Modified with Magnesium

**Scheme 29:**
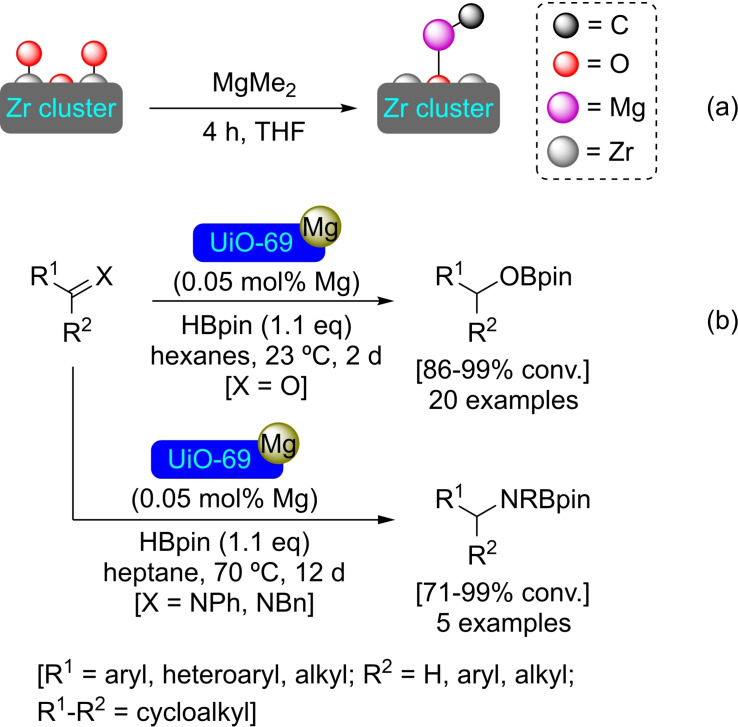
(a) Schematic coordination of the [Mg]@UiO‐69 prepared by SIM with MgMe_2_ (for simplicity hydrogen atoms are omitted), and (b) catalytic hydroborylation of C=O and C=NPh bonds with [Mg]@UiO‐69.

A metal‐organic framework based on the UiO‐69 topology (with zirconium clusters as nodes) has been prepared employing 2′′‐nitro‐[1,1′′:4′,1′′:4′′”,1′′′‐quaterphenyl]‐4,4′′′‐dicarboxylate (tphn) as ligand, for subsequent functionalization with magnesium. The prepared material has been treated with dimethylmagnesium, allowing the formation of MeMg−O‐ units at the nodes with methane release (Scheme 29a). This [Mg]@UiO‐69 has shown excellent catalytic activity in the hydroboration of carbonyl compounds and imines using pinacolborane. It has been postulated that the catalytic cycle proceeds with the formation of magnesium hydride as an intermediate.^[83]^ In addition, the catalyst has allowed the intramolecular hydroamination of 4‐pentenylamines (Scheme 29b).

### 3.7 Zr_12_(tpdc) Modified with Cobalt

**Scheme 30:**
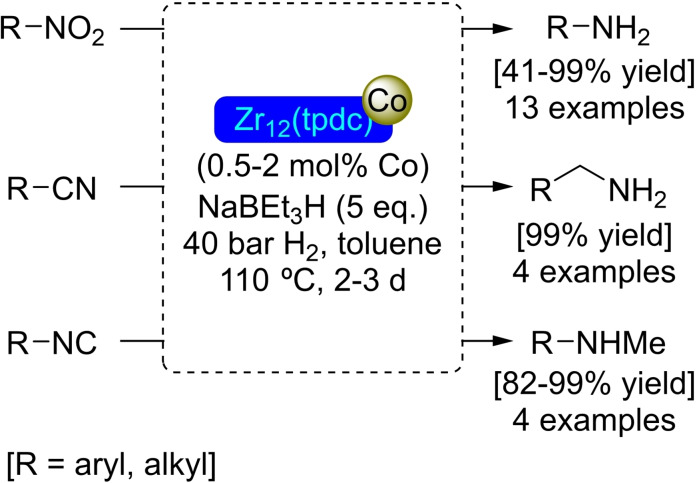
Catalytic reduction of nitro, nitrile and isonitrile derivatives with [Co]@Zr_12_(tpdc).

Modulation of a metal‐organic framework preparation conditions allows for the synthesis of different structures from the same starting components. Thus, a Zr_12_O_8_(μ_3_‐OH)_8_(μ_2_‐OH)_6_ node can be obtained, with the addition of the appropriate amount of water, instead of the more usual Zr_6_O_4_(μ_3_‐OH)_4_ by dimerization of the latter. In the presence of the ligand terphenyldicarboxylate (tpdc) the corresponding MOF, Zr_12_(tpdc), is formed, which has pores similar in size and shape to UiO‐68. The larger size of the nodes increases the molecular mass for the same type of structure, so the surface area per gram of MOF is smaller than in UiO‐68. The treatment of Zr_12_(tpdc) successively with (trimethylsilyl)methyl lithium and cobalt(II) chloride results in the metalation of the material at the nodes, incorporating about 11 cobalt atoms per node (of Zr_12_). The new [Co]@Zr_12_(tpdc) material has been transformed into an active catalyst for the reduction of nitroarenes, nitriles, and isocyanides to the corresponding amines by treatment with sodium triethylborohydride (Scheme 30), forming cobalt hydride species on the nodes.^[84]^

### 3.8 Zr(mtbc) Modified with Cobalt

**Scheme 31:**
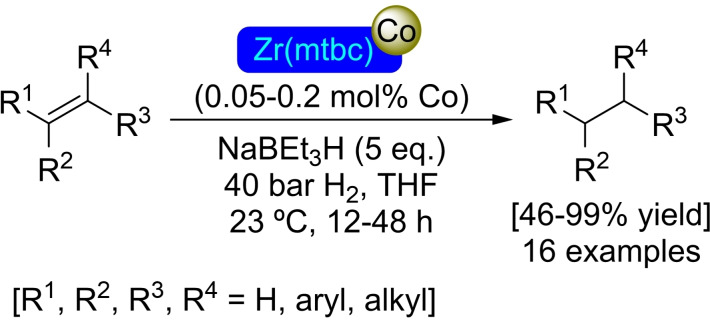
Catalytic hydrogenation of olefins with [Co]@Zr(mtbc).

The use of the tetradentate ligand 4‐[4‐[tris[4‐(4‐carboxyphenyl)‐phenyl]methyl]phenyl]benzoic acid (mtbc) in combination with zirconium results in the preparation of the MOF, Zr(mtbc), in which two distinct types of nodes are present. In addition to the usual Zr_6_ node, a second type of node with eight zirconium atoms [Zr_8_(μ_2_‐O)_8_(μ_2_‐OH)_4_] is present in a 1 : 3 ratio to the regular Zr_6_O_4_(OH)_4_ nodes. As in other UiO‐type MOFs, Zr_6_ nodes define octahedral pores, while Zr_8_ nodes define cubic pores. Regarding its modification, Zr(mtbc), has been treated with butyllithium to deprotonate the ‐OH groups in the nodes, for subsequent binding of cobalt atoms by SIM with cobalt(II) chloride. Analogous to other MOFs on which cobalt has been deposited, the [Co]@Zr(mtbc) treatment with sodium triethylborohydride results in the formation of cobalt hydrides that are active catalysts in different reductive transformations. Indeed, the activated material is effective in the hydrogenation of olefins (including tri‐ and tetrasubstituted), carbonyls, and imines (Scheme 31). The catalyst can be recycled up to 5 times, with no loss of activity or structural degradation.^[85]^

### 3.9 NPF‐520 Modified with Iron

**Scheme 32:**
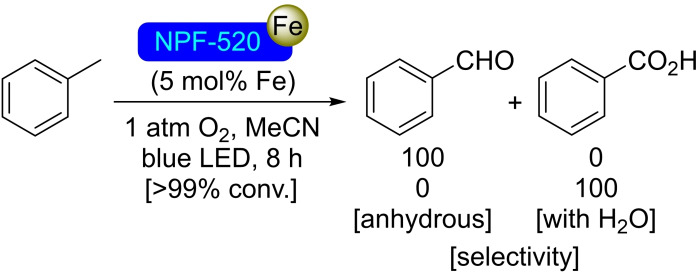
Catalytic selective oxidation of toluene with [Fe]@NPF‐520.

NPF‐520 is a novel MOF containing the recently discovered [Zr_9_O_9_(OH)_6_(H_2_O)_6_] node in combination with 4,4′,4′′,4′′′‐([9,9′‐bicarbazole]‐3,3′,6,6′‐tetrayl)tetrakis(3‐methylbenzoic acid) linkers in an ith topology. Two distinct zirconium nodes are present, one bridged by four *μ_3_*‐O_2_^−^/OH^−^, three linker molecules and a terminal H_2_O, with the second being bonded by two linkers and seven *μ_3_*‐O_2_^−^/OH^−^ groups. Not present in common 12‐connected Zr_6_ nodes, the terminal ‐OH/ H_2_O groups offer additional anchoring points towards postmodification. In this sense, NPF‐520 was metalated with iron via SIM with FeCl_3_, resulting in the even incorporation of ca. 3.1 Fe atoms per node throughout the material, with retention of crystallinity but significant decrease in the specific surface area (2500 m^2^/g from 3500 m^2^/g) and a slight decrease in pore size. The so‐obtained material, with near‐visible light absorption, was assayed in the photocatalytic oxidation of toluene under blue light and compared with other Fe‐loaded MOFs. [Fe]@NPF‐520 shows complete selectivity towards benzaldehyde in anhydrous conditions (Scheme 32), and much higher performance than iron‐modified UiO materials due to enhanced light harvesting and energy transfer from the bicarbazole ligand. The unmodified NPF‐520, on the other hand, is completely inactive in this transformation.^[86]^

### 3.10 MOF‐5 Modified with Rhodium

Terephthalic acid in combination with zinc(II) salts result in the formation of the historically significant MOF‐5, which in 1999 became the first MOF described with permanent porosity.^[87]^ The [Zn_4_O(CO_2_)_6_] cluster has a regular tetrahedron shape, with a single oxygen atom bonded to four zinc atoms with the edges capped by a CO_2_ group. The nodes are linked with terephthalate units resulting in a simple cubic network.^[10]^ While not as stable as zirconium MOFs, MOF‐5 has been assayed as support for catalytic systems. For instance, MOF‐5 has been modified with Rh(acac)(C_2_H_4_)_2_ by SIM, incorporating 1 wt % of rhodium in the material. This [Rh]@MOF‐5 has been reported as an efficient catalyst in the hydroformylation of butene, with high conversion (96 %) and, unlike their Zr counterparts, excellent selectivity towards pentanal (86 %).^[77]^ [Rh]@MOF‐5 was shown to be able to stabilize CO molecules without strong binding, allowing the catalytic cycle to proceed smoothly.

### 3.11 MOF‐808

Trimesate (btc) as a linker in combination with zirconium clusters is used for the preparation of MOF‐808. This material has tetrahedral cages with the nodes in the vertices and the linkers (btc) in the faces.

**Scheme 33:**
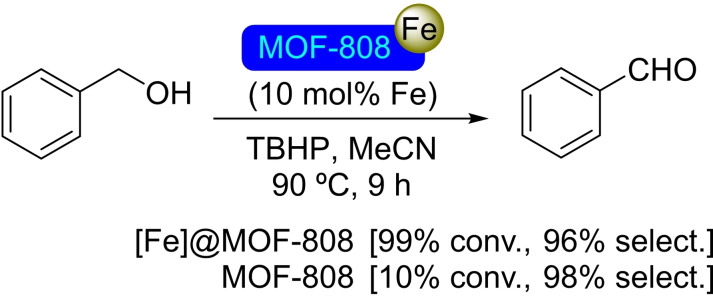
Catalytic oxidation of benzyl alcohol with [Fe]@MOF‐808.

**3.11.1. Iron modification**. Iron(III) acetylacetonate has been employed for the solvothermal deposition of iron(III) catalytic sites at the nodes of MOF‐808. Iron is incorporated in a ratio of 0.86 atoms per node, partially occupying the pore space, [Fe]@MOF‐808 has resulted active as a catalyst for the oxidation of benzyl alcohol to benzaldehyde in the presence of *tert*‐butyl hydroperoxide (Scheme 33). The selection of the solvent (mixture of acetonitrile/cyclohexane) was found to be crucial to avoid the overoxidation of the product aldehyde to the corresponding acid.^[88]^

**3.11.2. Palladium modification on MOF‐808 based on hafnium**. As other zirconium MOFs, MOF‐808 based on hafnium can be prepared using the same synthetic protocol, employing HfOCl_2_⋅8H_2_O, instead of a zirconium salt. This material has been used for palladium deposition by treating with aqueous solutions of phosphoric acid or sulfuric acid to incorporate phosphate or sulfate moieties, which help stabilize palladium(II) single‐sites and avoid the formation of nanoparticles. Thus, MOF‐808(Hf‐PO_4_) and MOF‐808(Hf‐SO_4_) were subsequently treated with a palladium(II) acetate solution to metalate the nodes. Phosphate modification seemed more effective at this, as in the oxidative Heck reaction between 2‐phenylphenol and ethyl acrylate, [Pd]@MOF‐808(Hf‐PO_4_) showed better catalytic activity than the [Pd]@MOF‐808(Hf‐SO_4_). Although initially successful, both systems deactivated after several hours of reaction with conversions of 50 % and 10 % to the coupling product, for [Pd]@MOF‐808(Hf‐PO_4_) and [Pd]@MOF‐808(Hf‐SO_4_) respectively, due to the eventual formation of inactive palladium nanoparticles. As proof‐of‐concept, this approach could potentially be further developed to increase stability of the Pd(II) single‐sites by grafting other ligand molecules.^[89]^

#### 3.11.1 HUST‐1 Modified with Nickel

**Scheme 34:**
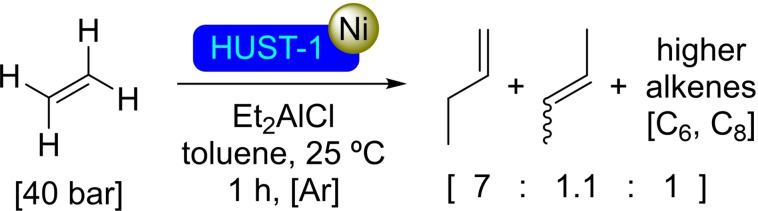
Catalytic dimerization of ethene with [Ni]@HUST‐1.

HUST‐1 is based on zirconium clusters employing 4,4′‐(4‐amino‐4*H*‐1,2,4‐triazole‐3,5‐diyl)dibenzoate as the linker. This ligand features a V‐shaped dicarboxylate design, which can improve stability by balancing flexibility and rigidity. As in all other cases, the presence of zirconium clusters with hydroxyl groups allows for straightforward post‐modification by SIM of HUST‐1 with solutions of different nickel(II) salts [i. e., chloride, bromide, nitrate, acetate, and oxalate] resulting in the incorporation of nickel ions in the material. Different coordination modes have been described in this modified material since nickel can be connected to the nodes but also to the triazole units present in the linkers. The [Ni]@HUST‐1 has been tested in the dimerization of ethylene, showing better activity and selectivity towards the formation of but‐1‐ene compared with [Ni]@UiO‐67, mostly due to the larger pore size of the former (Scheme 34).^[90]^

#### 3.11.2 PCN‐222(Fe) Modified with Iron

**Scheme 35:**
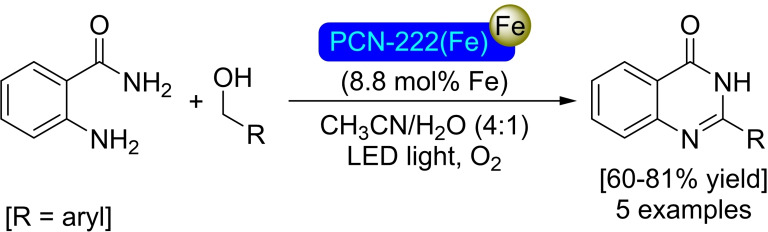
Photo‐oxidative catalytic reaction of benzyl alcohols and 2‐aminobenzamide with [Fe]@PCN‐222(Fe).

The iron‐porphyrin complex 5,10,15,20‐tetrakis(4‐carboxyphenyl)porphyrin‐iron(III) has been employed in combination with zirconium for the preparation of the biomimetic PCN‐222(Fe) MOF. In a recent work, the content of iron within this structure was increased further by SIM of PCN‐222(Fe) with an iron(III) chloride solution forming bimetallic Zr_6_Fe_2_ nodes. The post‐modified [Fe]@PCN‐222(Fe) has been revealed as an effective photocatalyst for the generation of oxygen reactive radicals, with the iron incorporated in the nodes improving the efficiency of electron‐hole pair separation. The same iron species also provide the material with increased Lewis acidity. This bifunctional character allowed [Fe]@PCN‐222(Fe) to mediate the challenging aerobic photo‐oxidative coupling of benzylic alcohols and 2‐aminobenzamides, affording quinazolin‐4‐ones in good yields under mild conditions (Scheme 35).^[91]^

#### 3.11.3 DUT‐5 Modified with Aluminum

**Scheme 36:**
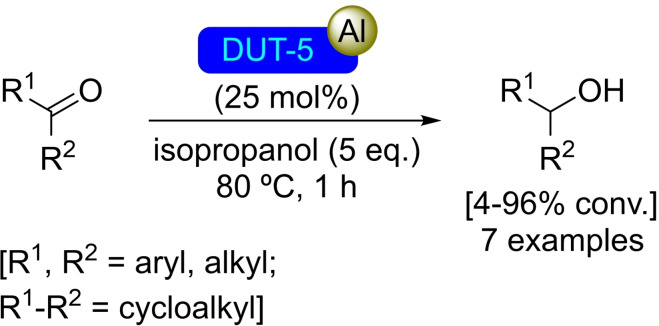
Catalytic Meerwein‐Ponndorf‐Verley oxidation with [Al]@DUT‐5.

DUT‐5 consists of chains formed by aluminum coordination octahedra linked by 4,4′‐biphenyldicarboxylate (bpdc) ligands. This MOF was first synthesized in 2009, by Senkovska's group, *via* a solvothermal approach.^[92]^ The material has a high permanent porosity, with a specific pore volume of 0.81 cm^3^/g, which makes it a good candidate for gas storage and compound adsorption applications. DUT‐5, although presenting sites with lower Lewis acidity than other materials such as UiO‐66 and UiO‐67, can be successfully metalated with Al active sites using trimethylaluminum. This [Al]@DUT‐5 material is active towards the reduction of aldehydes and ketones by Meerwein‐Ponndorf‐Verley reaction (Scheme 36), similar to the aluminum catalyst supported on UiO‐67. In contrast however, [Al]@DUT‐5 has shown higher tendency to deactivation upon recycling, in lieu of its lower intrinsic stability.^[67]^

#### 3.11.4 MIL‐101

The MIL series is another staple of MOFs, with a relevance similar to that of UiO materials. Also based in benzenedicarboxylate linkers, the use of trivalent metal centers, such as iron(III) or Cr(III) results in materials with enhanced stability and large pores. The MIL‐101 based on iron, MIL‐101(Fe), is particularly popular, as it is easily prepared by solvothermal synthesis using an iron(III) salt and terephthalate linkers.^[93]^

**3.15.1. Copper modification**. SIM of MIL‐101(Fe) in a solution of copper(I) chloride, and subsequent evaporation to dryness resulted in the formation of [Cu]@MIL‐101(Fe). This treatment resulted in the formation of copper‐iron oxide (CuFeO_x_) within the MOF structure. This mixed oxide causes changes in the electronic structure of the material, significantly altering its catalytic properties through enhanced electronic transfer and better coordinative capabilities. This effect was proved in the oxidative detoxification of organic pollutants, such as bisphenol A, with peroxymonosulfate, which showed up to 14‐fold increase in performance. A dual radical/non‐radical pathway was proposed, enabled by the electronic properties the CuFeO_n_ species.^[94]^

**3.15.2. Rhodium modification**. MIL‐101(Fe) has been metalated by solvothermal reaction with Rh(acac)(C_2_H_4_)_2_ to provide a supported catalyst [Rh]@MIL‐101 containing ca.1 wt % of rhodium. The resulting [Rh]@MIL‐101 catalyzed the hydroformylation of butene with high conversion (96 %) and fair selectivity (77 %) towards the formation of pentanal.^[77]^

#### 3.11.5 MIL‐125

MIL‐125 is a highly porous MOF is formed from titanium‐oxo‐hydroxo clusters [Ti_8_O_8_(OH)_4_] and terephthalate linker. This material, which has a pseudocubic arrangement with two types of cages, presents high thermal stability and, interestingly, intrinsic photochemical properties derived from the titanium clusters, thus being a promising candidate for multifunctional catalysis by post‐modification.^[95]^

**Scheme 37:**
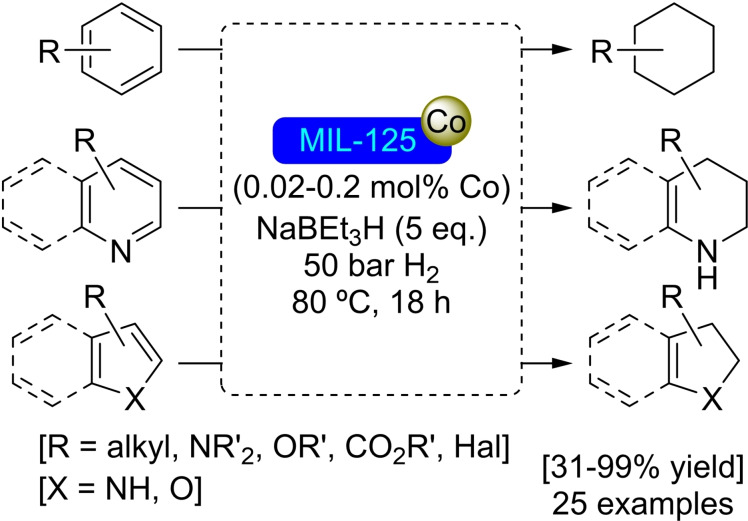
Catalytic hydrogenation of arene and heteroarene compounds with [Co]@MIL‐125.

**3.16.1. Cobalt modification**. MIL‐125 has been postmodified by subsequent treatment with (trimethylsilyl)methyllithium and cobalt(II) chloride, resulting in [Co]@MIL‐125 with a ratio of 0.7 atoms of Co per node. As observed with other cobalt‐modified MOFs, the reaction of [Co]@MIL‐125 with sodium triethylborohydride provides a supported cobalt hydride, which is catalytically active in reduction reactions. Thus, this system can effectively mediate the hydrogenation of a variety of arene and heteroarene compounds (Scheme 37).^[96]^

**Figure 8:**
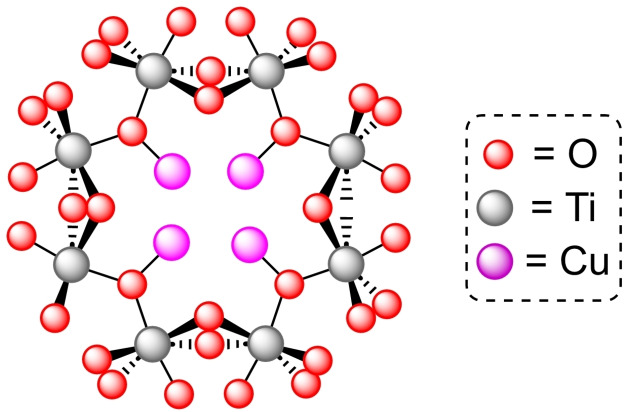
Model of the node structure of MIL‐125 modified with copper. For simplicity hydrogen atoms are omitted.

**3.16.2. Copper modification**. The preparation of MIL‐125‐NH_2_ is achieved through the same solvothermal process used for the parent structure, employing 2‐aminoterephthalate as linker instead of terephthalate. This material was treated with an excess of (trimethylsilyl)methyllithium and different amounts of [(CH_3_CN)_4_Cu]BF_4_ to prepare [Cu]@MIL‐125‐NH_2_ with a maximum of 4 copper(I) centers per node (Figure 8). This supported catalyst is active in the reduction of CO_2_ in the presence of H_2_, exhibiting high selectivity towards ethylene (>95 %). Noteworthy, the titanium clusters not only stabilize of the copper(I) centers, avoiding the formation of copper(0) nanoparticles, but the distance between copper atoms is ideal to generate C2 products (i. e. ethanol). As the titanium(IV) in nodes can efficiently catalyze ethanol dehydration to afford ethylene under the reaction conditions, this is a great example of a synergistic effect between the parent structure and the modification inserted.^[97]^

## 4 Modification of Nodes with Organic Molecules, Salts and Complexes

Different organic molecules, salts and complexes have been supported to well‐defined metal‐organic frameworks, mainly by SALI. As with metal modification, the species inserted provide the materials with enhanced or completely different catalytic activity. In this section, we have compiled a series of synthetic transformations promoted by MOFs modified with organic molecules, salts and complexes, which are discussed in the following subsections.

### 4.1 NU‐1000

As commented previously, NU‐1000 has been widely studied among the zirconium‐based metal‐organic frameworks, due to its straightforward preparation and well‐defined characteristics.^[31]^ To activate the NU‐1000 after a modulated synthesis with a carboxylic acid the material is treated with a mixture of DMF and HCl (aq.), which results in the removal of node‐blocking modulators, formate ions, aqua and/or hydroxo terminal‐ligands. The use of DMSO, rather than DMF, affords a material free of formate.^[98]^ These postsynthetic treatments could be of interest for further modifications.

**Scheme 38:**
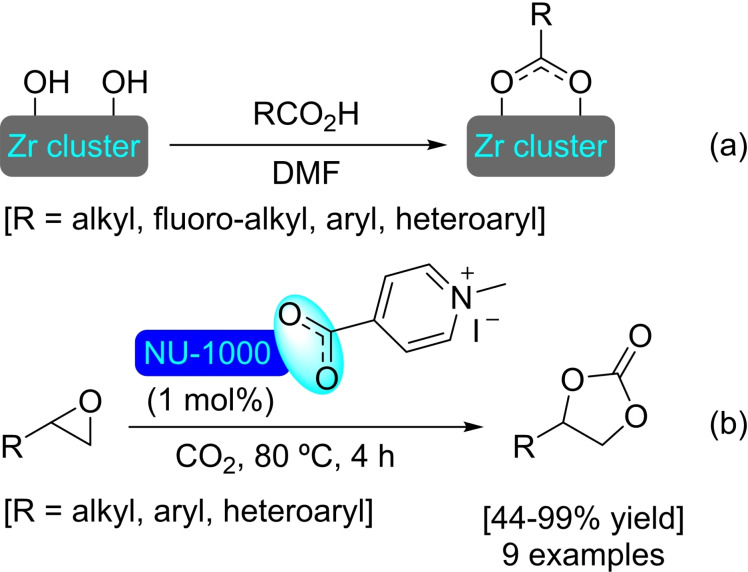
(a) Proposed model of modified NU‐1000 with carboxylates, and (b) ring‐opening of epoxides with CO_2_ using functionalized MOF as catalyst.

**4.1.1. Modification with carboxylates**. The modification of NU‐1000 with carboxylate‐bearing molecules is probably the most obvious, owing to the well‐established use of carboxylate modulators in the synthesis of Zr MOFs. It is also very convenient, as the carboxylate group is very stable and readily available in a vast variety of relevant organic compounds. The functionalization is very simple, based on an acid‐base interaction between the hydroxyl groups in the nodes and the carboxylate group. In recent literature, plenty of examples of such modification on NU‐1000 have been reported. A SALI protocol has been used to insert perfluoroalkyl carboxylates (2 to 9 carbon chains), netting about 3–4 molecules per node (Scheme 38a). This type of modified MOFs presented positive synergistic activity in the adsorption of CO_2_ due to the presence of C−F dipoles,^[99]^ and has shown to be an effective approach for bringing about stability,^[100]^ as well as regulating transport and diffusivity within NU‐MOFs.^[101]^ Similarly, the CO_2_ adsorption of NU‐1000 can also be increased via the incorporation of organic motifs, such as Fmoc‐triglycine and 2,6‐diacetylamido‐4‐carboxypyridine, that feature partial charge distribution for better interaction with the guest molecule.^[102]^ Showcasing the scope of this type of modification, different alkyl and aryl carboxylic acids bearing functional groups, such as halide, acetylene, hydroxyl, thiol, amine, azide and aldehyde (Scheme 38a), followed by late‐stage functionalization of the substituent moieties (e. g. triazole formation by ‘click reaction’ of the alkyne, or imine formation with the aldehyde) were inserted in NU‐1000 in a recent report.^[103]^ In addition, 2‐, 3‐, and 4‐pyridinecarboxylic acids have been implanted within the MOF, then subsequently reacted with alkyl halides (i. e. methyl iodide, butyl iodide, butyl bromide, 3,3,4,4,5,5,6,6,6‐nonafluorohexyl iodide) to form the corresponding supported pyridinium salts. The pyridinium‐MOF salts showed improved activity over the pristine structure in the adsorption and valorization of CO_2_ via reaction with epoxides to afford cyclic carbonates (Scheme 38b).^[104]^ This type of modification not only brings catalytic properties by itself but can also help regulate the deposition of other catalytic species in the structure. In this sense, naphthalene‐2,6‐dicarboxylic acid has been incorporated in NU‐1000 to block small cavities by bridging two nodes, which was exploited to induce the subsequent deposition of metallic species [e. g. cobalt(II)] in specific sites oriented toward mesoporous channels.^[39]^

Beyond catalysis, different molecular units have been introduced in NU‐1000 to prepare highly task‐specific materials and delivery vehicles. Of particular interest are carboxylate‐based drug molecules, such as ketoprofen, nalidixic acid, and levofloxacin, which have been loaded in NU‐1000 and then released under simulated body fluid conditions (reverse‐SALI), showcasing the potential of MOFs in drug delivery.^[105]^ 3,5‐Dinitrobenzoic acid^[106]^ and ferrocenecarboxylic acid^[107]^ have also been inserted in this MOF and combined with β‐cyclodextrin to increase the conductivity of the material, targeting applications in electrochemistry.^[108]^ The modification with photoactive [6,6]‐phenyl‐C_61_‐butyric acid (PCBA), which incorporates 2 PCBA units per node, enhances the singlet oxygen production of the material, allowing the photocatalyzed detoxification of mustard gas and similar sulfide‐based nerve agents by oxidation to the corresponding sulfone.^[109]^ Similarly, photoactive NU‐1000 has been prepared by incorporation of boron‐dipyrromethane (BODIPY) carboxylates, showing greatly increased activity in the oxidation of 1,5‐hydroxynaphthalene to juglone.^[110]^ The modification with 5(6)‐carboxynaphthofluorescein (CNF) resulted in a material with halochromic properties, and has potential application as a visual pH‐indicator.^[111]^ Another modification is the incorporation of mercaptoacetic acid (MAA) to the NU‐1000 nodes resulting in a colorimetric detector of heavy metal ions, such as mercury(II), in aqueous solutions.^[112]^ The insertion of 2‐mercaptobenzoic acid in the nodes allowed the deposition of silver(I) ions which can be reduced to form active silver nanoparticles, with increased stability in the structure.^[113]^ The insertion of a Gd(III) complex with a terminal carboxylic acid in the Zr_6_ nodes resulted in a material for magnetic resonance imaging (MRI) contrast.^[114]^ 6‐[(2,2′:5′,2′′:5′′,2′”:5′”,2′′′′‐quinquethiophen)‐3′′‐yl]hexanoic acid has been incorporated to NU‐1000 generating a material with electronic conductivity and only a 25 % reduction of the overall porosity.^[115]^ The incorporation of a semirotaxane to a microcrystalline sample of NU‐1000 has also been achieved, using the node as cap for the rotaxane.^[116]^ The redox‐active rotaxane, inserted with an average of one rotaxane component per cluster, has provided an approach for the design of solid‐state molecular machines supported within microporous structures.

**Scheme 39:**
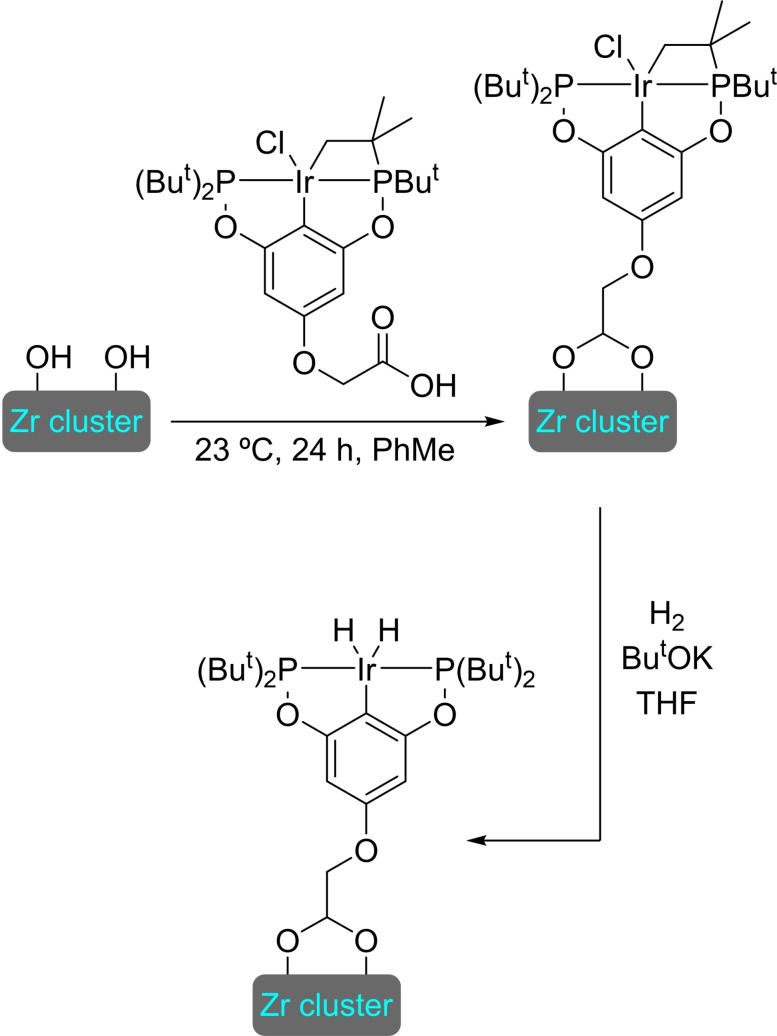
Proposed model of modified NU‐1000 with iridium complex, prepared by SALI, and subsequent activation.

Besides purely organic compounds, the use of metallic complexes that present a carboxylic moiety allows to insert well‐known catalysts in the MOFs, affording a myriad of heterogeneous catalysts. This approach is very interesting, as the interior of the MOF is often sufficiently spacious to offer a pseudo‐homogeneous medium in which to carry out the reaction, meaning the intrinsic catalytic activity of the complex should not change significantly. In many cases, an increase in performance has been observed, probably due to the higher effective concentration of reactants within the structure and much higher stability of the complexes protected within the confines of the MOF. Depending on the structure used, size selective catalysis is also enabled, all this showing the incredible potential of such modification in MOF chemistry. Following this trend, an iridium(III) pincer complex was prepared bearing a carboxylic acid moiety to carry out a post‐modification of NU‐1000. Thus, 5‐(carboxymethoxy)‐1,3‐bis(di‐*tert*‐butylphosphite)benzene iridium(III) hydride was incorporated to the MOF in solution (Scheme 39), resulting in 0.8 to 1 iridium‐complexes grafted per node by Ir/Zr ratio as measured by ICP. The modified material resulted active in the hydrogenation of alkenes (i. e. dec‐1‐ene and styrene in solution, and ethene in gas phase). The supported complex proved to be more active and stable than its homogeneous counterpart, highlighting the advantages mentioned earlier.^[117]^ Similarly, a rhodium(III) complex with 2,2’‐bipyridine‐5‐carboxylic acid as ligand has been successfully immobilized at the zirconium nodes via SALI, with the resulting material proving to be useful in the electrocatalytic regeneration of NADH during the conversion of pyruvate into l‐lactate.^[118]^

**Scheme 40:**
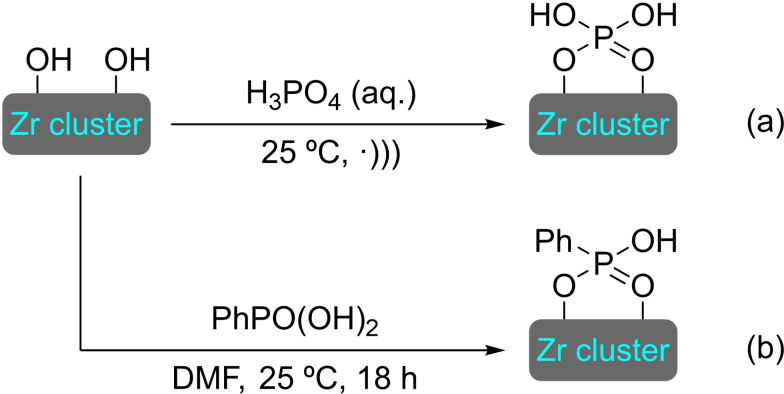
Plausible model and protocol for modification by SALI of NU‐1000 with (a) phosphate, and (b) phenylphosphonic acid.

**4.1.2. Modification with phosphates and phosphonates**. Phosphorous based compounds such as phosphate^[119]^ and phenylphosphonate,^[120]^ have been grafted onto the nodes of NU‐1000 by SALI (Scheme 40). This type of ligands is incorporated employing mild conditions due to the strong bonds formed with high‐valent transition metals, with a general upper limit of 4 ligands per node before the phosphorous based ligands begin to negatively affect the structural integrity of the material by displacement of the constitutive carboxy ligands of the MOF. This way, a phosphoric acid aqueous solution was used to incorporate phosphate species, resulting in poisoning of the strong Lewis‐acid sites in the nodes, effectively modulating the acidity of the material. The phosphate‐modified NU‐1000 was tested as catalyst in the synthesis of 5‐hydroxymethylfurfural (HMF) from glucose with promising results in terms of activity and selectivity. By carefully controlling the amount of phosphoric acid incorporated in the MOF, a material with good balance between activity and selectivity towards the glucose‐to‐fructose isomerization and fructose‐to‐HMF dehydration was obtained.^[119]^

**Scheme 41:**
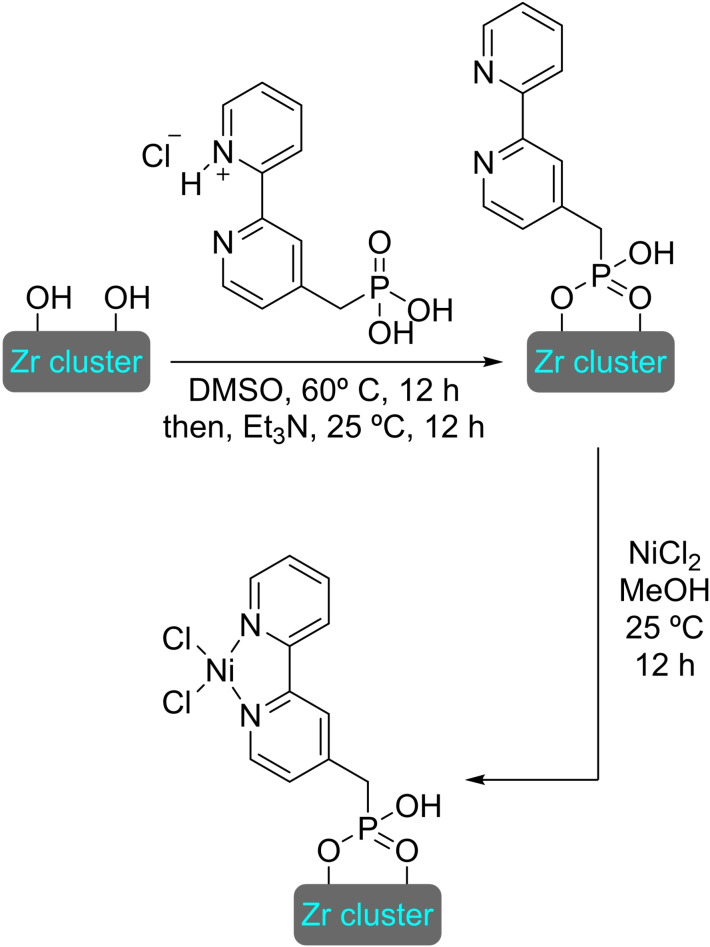
Proposed model and protocol for modification of NU‐1000 with a phosphonic acid as ligand for nickel complex.

Following the same strategy, NU‐1000 has been treated with a DMSO solution of 5‐methylphosphonate‐2,2’‐bipyridine resulting in the incorporation of this motif to the nodes of the MOF.^[121]^ The bipyridine ligand grafted to the material was subsequently employed to form nickel(II) complexes, with a loading of 1.1 Ni(II) per node (Scheme 41). The resulting supported catalyst was activated with diethylaluminum chloride to be employed in the dimerization of ethylene to form but‐1‐ene. The activity of the reported [bpyNi(II)]@NU‐1000 was observed to be a few orders of magnitude higher than the corresponding homogeneous analogue, while being possible to reuse it due to the immobilization‐induced stabilization extending the useful life of the complex.

### 4.2 UiO‐66

**Scheme 42:**
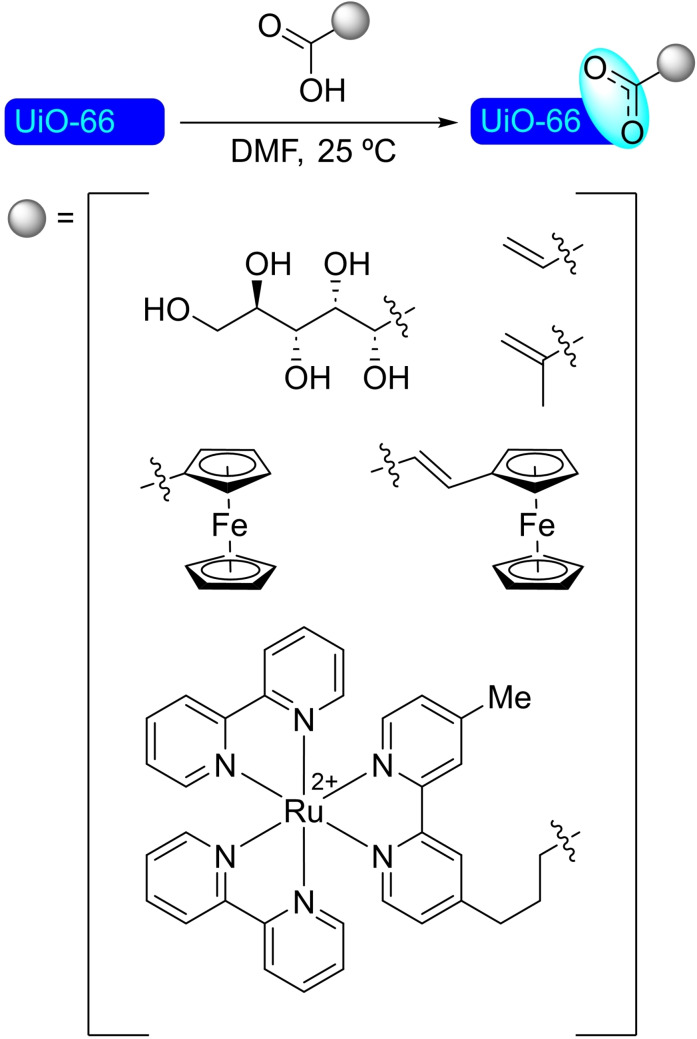
Modification of UiO‐66 by SALI with different carboxylic acid derivatives.

**4.2.1. Modification with carboxylates**. Pristine UiO‐66 was submerged into a solution of d‐gluconic acid allowing the incorporation of free‐hanging hydroxyl moieties (Scheme 42), thus resulting in a material exhibiting both Lewis acidity and hydrogen bond donor capabilities. This synergistic modification increased its CO_2_ uptake ability while allowing its use as a cooperative catalytic system in the cycloaddition of CO_2_ with epoxides to form cyclic carbonates.^[122]^ Similarly, ferrocenecarboxylic acid and 3‐ferrocenylpropenoic acid have been readily inserted into the structure of UiO‐66 via SALI (Scheme 42), with an average 1.3 molecules grafted per node. While UiO‐66 is not electrochemically active, the materials functionalized with ferrocene are, with the, electroactivity mostly attributed to the ferrocene moieties attached at or near the external surface of the MOF.^[123]^ Other metal complexes, such as Ru(bpy)_2_(mcpbpy)^2+^ [bpy=2,2’‐bipyridine, mcpbpy=4‐(4’‐methyl‐(2,2’‐bipyridin)‐4‐yl)butanoic acid], have been grafted onto the coordinatively unsaturated zirconium nodes (Scheme 42), giving a material with properties adequate for its use as an electrochemiluminescence probe.^[124]^ On another note, acrylic acid has been employed to modify the UiO‐66 structure, forming a cross‐linked metal‐organic framework (Scheme 42). This altered material has been combined with Arabic gum to form a hydrogel, with potential applications for the remediation of organic pollutants. The formation of the hydrogel resulted in a five‐fold increase in elastic modulus over the MOF.^[125]^

As the use of dichloroacetic acid (DCA) as modulator during the preparation of UiO‐66 provided highly porous, well‐dispersed nanoparticles of material. As proof of concept, [DCA]@UiO‐66 has been post‐synthetically loaded with different drugs with carboxylic moieties, such as ibuprofen and α‐cyano‐4‐hydroxycinnamic acid, observing an inverse correlation between the pK_a_ of the acid and incorporation. Interestingly, combinations of different drugs have been loaded onto the material, targeting synergistic increases of activity within a single material.^[126]^ Similar strategies may be of interest to prepare materials with cooperative units for catalysis, in a way not dissimilar to the d‐gluconic acid‐modified material described above.

**4.2.2. Modification with phosphates and phosphonates**. The phosphonic acid moieties present in alendronic acid have been employed to modify the structure of UiO‐66.^[126]^ In this way, a mitochondrial targeting molecule [(3‐carboxypropyl)triphenylphosphonium bromide] and a tumoral targeting molecule (folic acid) have been chemically linked to alendronate to incorporate these molecules to the structure of a porphyrin‐cobuilt UiO‐66 (i. e. UiO‐66 formed using a 0.07 : 1 mixture of meso‐tetra(4‐carboxyphenyl)porphyrin and terephthalic acid linkers). The functionalized material is interesting for photodynamic therapy (PDT), where it would massively accumulate into a tumor, localizing into the mitochondria, and the presence of porphyrin units would induce the production of cytotoxic singlet oxygen by red‐light irradiation.^[127]^ In addition, the post‐synthetic loading of UiO‐66 with antibiotics, such as fosfomycin, has been described due to the interaction of the phosphate group with the nodes.^[128]^

**Scheme 43:**
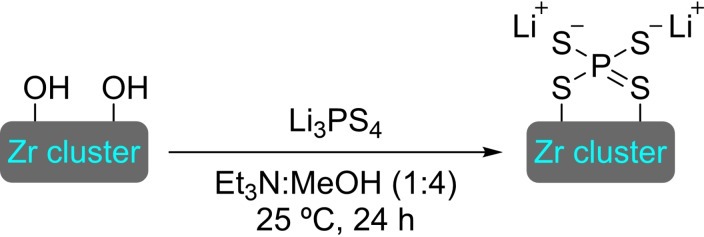
Modification of UiO‐66 by SALI with thiophosphate.

UiO‐66 has been also employed to stabilize lithium thiophosphate, which bound to the open sites of the zirconium nodes, via SALI (Scheme 43), resulting in a material with promising application in lithium‐sulfur batteries.^[129]^ An iron(III) doped UiO‐66 has been modified by impregnation in a solution of octadecylphosphonic acid giving a hydrophobic material targeting potential use in biphasic redox processes. Indeed, this modified MOF was used to catalyze the oxygen reduction to hydrogen peroxide in a two‐phase system (water/benzyl alcohol). The presence of iron(III) ions allowed the visible‐light absorption acting as an electron donor for O_2_, whereas the hydrophobic moieties provided a spatial separation inhibiting the decomposition of the generated H_2_O_2_ by the same material.^[130]^

**Scheme 44:**
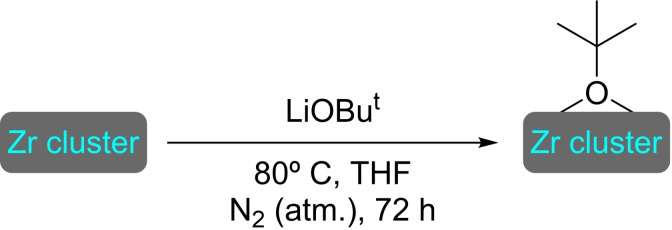
Modification of UiO‐66 by SALI with an alkoxide.

**4.2.3. Modification with alkoxides**. UiO‐66 can be functionalized with lithium *tert*‐butoxide by first dehydrating the material under dynamic vacuum and subsequent exposure to a solution of the alkoxide (Scheme 44). The structure of the material remains intact upon grafting, resulting in a solid with potential application for lithium‐based batteries.^[131]^ Similar treatment of a cobuilt UiO‐66 using 1,4‐benzenedicarboxylate and 2‐amino‐1,4‐benzenedicarboxylate (3 : 1 ratio) resulted in a material with catalytic activity to hydrolyze chemical warfare agents, such as soman and sulfur mustard.^[132]^

### 4.3 MOF‐74 Modified with Amines

The combination of 2,5‐dihydroxybenzene‐1,4‐dicarboxylic acid (dobdc) with a divalent metal ion (e. g. Mg, Zn, Cu, Co, Ni, Fe and Mn) gives rise to the MOF‐74 series of materials, which have hexagonal channels of *ca*. 12 nm. Metal centers coordinate with five oxygen atoms from the linker units, generating a Lewis acidic open metal site. Besides the intrinsic catalytic activity of open metal sites, this also results in more flexibility regarding composition. Thus, great interest has been shown towards the preparation of mixed‐metal MOF‐74 with synergistic effects, as well as using analogue linkers, such as biphenyl, *p*‐terphenyl, naphthalene, and anthracene, giving the same topology with a variation in the size of pores and surface areas.^[133]^

**Scheme 45:**
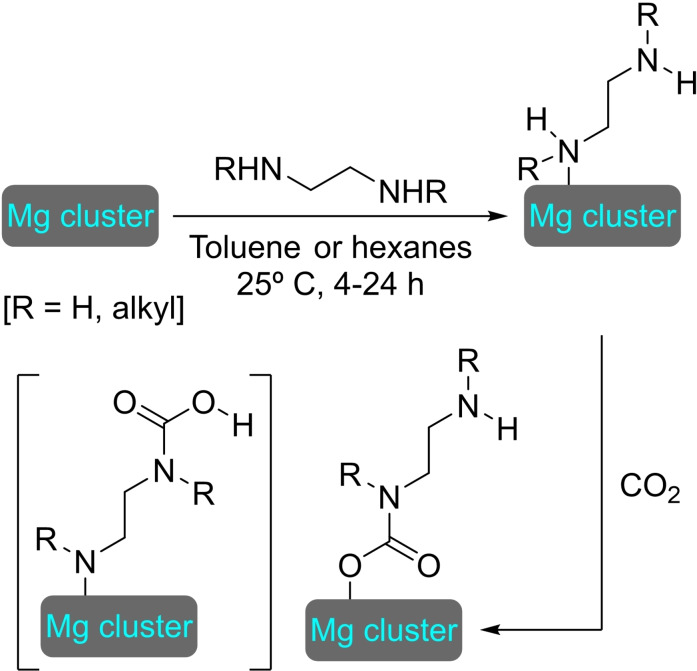
Modification of MOF‐74 by SALI with ethylenediamines, and its interaction with CO_2_.

The post‐modification of MOF‐74(Mg) with molecules containing multiple amino groups, such as ethylenediamine,[^134^, ^135^, ^136^] tetraethylenepentamine,[^137^, ^138^] or hydrazine,^[139]^ results in the preparation of a material with improved carbon dioxide adsorption properties at low partial pressures of this gas (Scheme 45). One amino group anchors the molecule to the Mg atoms while the other interacts with CO_2_, reducing its mobility, which is exploited for the separation of CO_2_/H_2_ mixtures.^[140]^ Indeed, the analogous MOF based on magnesium and 4,4’‐dihydroxi‐3,3’‐biphenyldicarboxylate (dobpdc) has been modified with *N*,*N*’‐dimethylethylenediamine[^141^, ^142^] or *N*,*N*‐dimethylethylendiamine^[143]^ by SALI with an excess of the diamine, likewise improving its carbon dioxide adsorption performance (Scheme 45).^[144]^ The analogue material with zinc has been also prepared and modified, obtaining similar results.^[141]^ Furthermore, a version of MOF‐74(Mg) using 1,5‐dihydroxinaphthalene‐2,6‐dicarboxylate as linker has been also prepared and altered with ethylenediamine, *N*,*N*’‐dimethylethylenediamine or piperazine following an analogous procedure, targeting the same type of application.^[145]^

### 4.4 MOF‐808

**4.4.1. Modification with carboxylates**. Ethylenediaminetetraacetic acid (EDTA) has been incorporated into the robust MOF‐808 by SALI in aqueous solution, resulting in the incorporation of two EDTA molecules per node. This functionalized material presents ultrahigh removal efficiency toward heavy metal ions,^[146]^ being effective against a broad‐spectrum of ions (i. e. soft Lewis acids, hard Lewis acids, and borderline acids) while also showing selective adsorption of SO_2_ over CO_2_ and N_2_ even at low SO_2_ partial pressure.^[147]^ As comparison, the inclusion of oxalic acid or thioglycolic acid rather than EDTA in the structure of MOF‐808 formed functionalized materials with inferior metal ions removal performance. Interestingly, the high coordinative efficiency of [EDTA]@MOF‐808 towards metal ions can be applied to prepare supported metal catalysts with controlled dispersion of active sites. This was proven by adsorption of palladium, resulting on a supported catalyst effective in the Suzuki cross‐coupling reaction.^[146]^

**Scheme 46:**
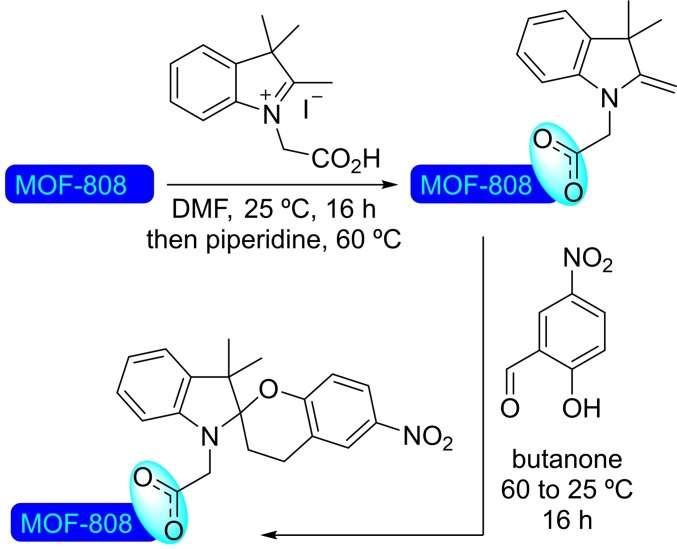
Modification of MOF‐808 by SALI with carboxylic acid, and subsequent transformation of the molecule linked.

As in most other cases, carboxylic acids are the most popular anchoring moieties regarding the modification of MOF‐808. This way, 2‐sulfo‐1,4‐benzenedicarboxylic acid has been bound onto the zirconium cluster of the MOF through the carboxylate moiety in position 1, as it is the preferred anchoring point due to its higher coordination ability. This modification results in a significant enhancement of the water adsorption capabilities and proton conductivity of the material.^[148]^ Besides direct insertion, a tandem anchoring/modification procedure can be used to insert more complex moieties. This way, 2‐[3’,3’‐dimethyl‐6‐nitrospiro‐(chromene‐2,2’‐indolin)‐1’‐yl]acetic acid has been inserted in the structure of MOF‐808 via a two‐step procedure: (a) MOF‐808 was treated with a solution of 1‐(carboxymethyl)‐2,3,3‐trimethyl‐3*H*‐indolium iodide, then (b) reacted with a solution of 2‐hydroxy‐5‐nitrobenzaldehyde, leading to the formation of the spiropyran compound in situ (0.5 units per node, Scheme 46). This functionalized MOF‐808 showed photoresponsive properties, targeting light‐modulated applications.^[149]^ This strategy is also valid for the preparation of hybrid materials, as shown by the modification of MOF‐808 with 4‐aminobenzoic acid with the hanging amino group being employed in the construction of a MOF‐supported covalent organic framework (COF). This represents a synthetic strategy in building covalently connected MOF‐COF structures with controlled morphologies leading to highly efficient task specific materials. This particular example was used for photocatalytic H_2_ evolution.^[150]^ On a different note, catechol‐benzoic acids (i. e. 2,3‐dihydroxybenzoic acid and 3,4‐dihydroxybenzoic acid) have also been inserted by SALI, using the catechol moiety as binding ligand for metals, such as copper, iron, manganese, mercury and nickel, focusing on different types of catalysis. Indeed, the copper‐functionalized material was successfully employed in the cycloaddition of benzyl azide and different aryl alkynes.^[151]^

**4.4.2. Modification with phosphates and phosphonates**. Methyl‐, ethyl‐ and vinylphosphonate have been incorporated to the structure of the MOF‐808 by SALI without framework collapse, as in this case, the lower acidity of these phosphonic derivatives makes them unable to displace the constitutive btc ligands. These functionalized materials have tested as adsorbents for the removal of uranium from wastewater.^[152]^ SALI of MOF‐808(Hf) in a solution of phosphoric acid resulted in the incorporation of phosphate units to the nodes, which has been employed to stabilize palladium species for catalytic oxidative Heck reactions.^[89]^ Analogously to UiO series of MOFs, MOF‐808 has also been employed to stabilize lithium thiophosphate at the open sites of the zirconium nodes via SALI. This material has promising application in lithium‐sulfur batteries.^[129]^

**Scheme 47:**
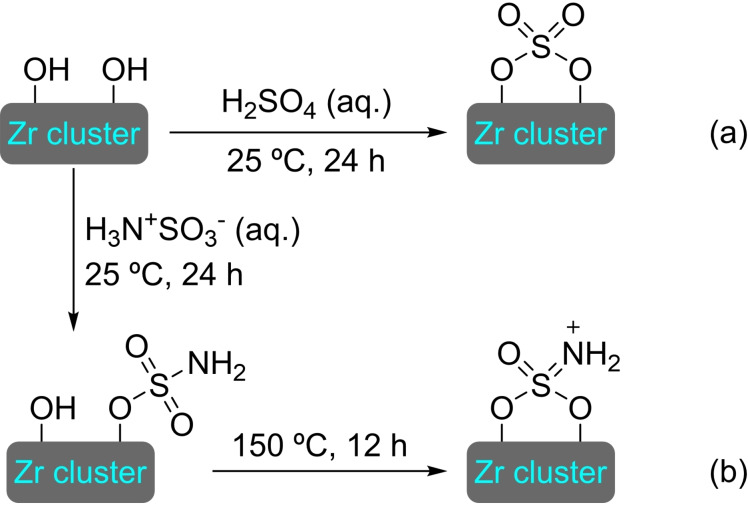
Proposed modification of MOF‐808 by SALI with (a) sulfuric acid, and (b) sulfamic acid.

**4.4.3. Modification with sulfates, sulfamates and persulfates**. Treatment of MOF‐808 with diluted aqueous sulfuric acid (0.05 M) resulted in the formation of a superacid sulfated MOF (Scheme 47).^[153]^ Similarly, a sulfamate‐functionalized material prepared analogously showed similar characteristics, previous activation at 150 °C (Scheme 47).^[154]^ The strong acidity and enhanced proton conductivity of these functionalized materials have been proven in catalytic reactions. The sulfate‐modified material exhibits good catalytic activity for the selective dimerization of isobutene, affording the two possible isomers were observed (2,4,4‐trimethylpent‐1‐ene and 2,4,4‐trimethylpent‐2‐ene) with a 4 : 1 ratio in favor of the terminal alkene while avoiding polymerization.^[155]^ MOF‐808 modified with sulfate units has also been used to stabilize palladium species for catalytic oxidative Heck reactions.^[89]^ Treatment of MOF‐808 with a diluted solution of sodium persulfate which coordinates in a bidentate fashion to a single metal node, results on a material loaded with an active peroxide precursor with potential applications as bactericide as well in oxidation reactions.^[156]^

**Scheme 48:**
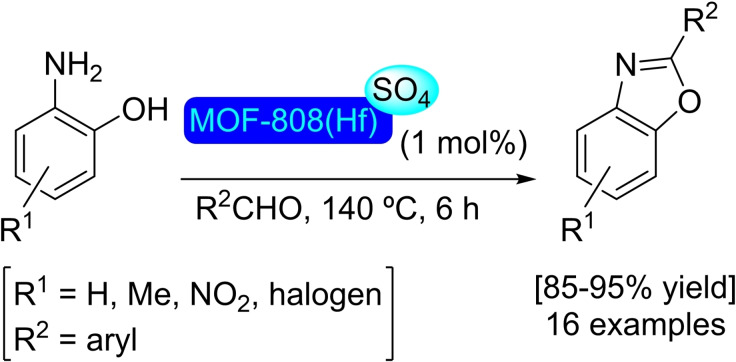
Solvent‐free synthesis of benzoxazoles catalyzed by [SO_4_]@MOF‐808(Hf).

MOF‐808(Hf) has also been modified by treatment with aqueous sulfuric acid (0.1 M), providing in a similar way a superacid material. The sulfated MOF was employed as an efficient and reusable (up to 5 times) catalyst in the reaction between benzaldehydes and 2‐aminophenols to synthesize 2‐arylbenzoxazoles (Scheme 48).^[157]^

**4.4.4. Modification with alkoxides**. A formate‐modulated MOF‐808 has been modified with methoxide by simply refluxing the material in methanol, resulting in the displacement of formate and residual DMF from the synthesis from the node, resulting in a significant change in the chemical properties of the material. Thus, the activated MOF‐808 showed much higher activity in the reduction of aldehydes and ketones by transfer hydrogenation using isopropanol as hydrogen source. Interestingly, this material was effective at reducing furfural and other related biomass‐derivatives as well.^[158]^

**4.4.5. Modification with amines**. MOF‐808 has been modified with triethylenediamine by vapor deposition after activation under helium at 150 °C. One of the nitrogen atoms interacts with a hydroxy group in the node while the other nitrogen is free to interact with different compounds. The functionalized material has been tested for removing chemical warfare agents, such as cyanogen chloride, achieving excellent results.^[159]^

**Scheme 49:**
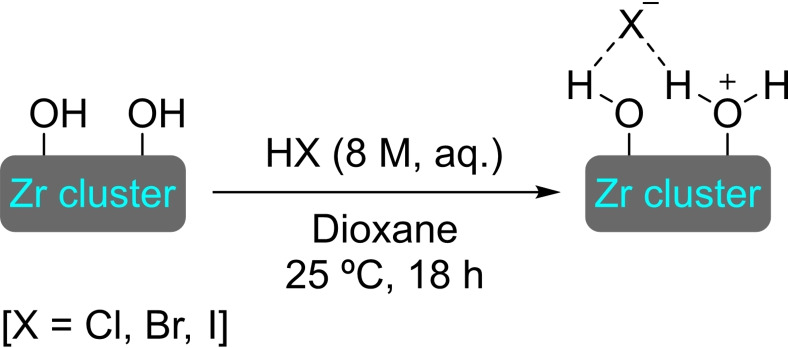
Modification of MOF‐808 by SALI with hydrogen halides.

**4.4.6. Modification with halides**. The treatment of MOF‐808 with dioxane solutions of aqueous HCl and HBr resulted in the incorporation of the corresponding halide (ca. 3.5 halide per node) in the material by hydrogen bond with the OH groups connected to the nodes (Scheme 49). The protocol can be also applied with HI (aq.), although with lower incorporation of iodide (ca. 2.3 iodide per node). The halide‐loaded material showed increased water uptake capacity and higher stability during its release.^[160]^

### 4.5 MIL‐100

**Scheme 50:**
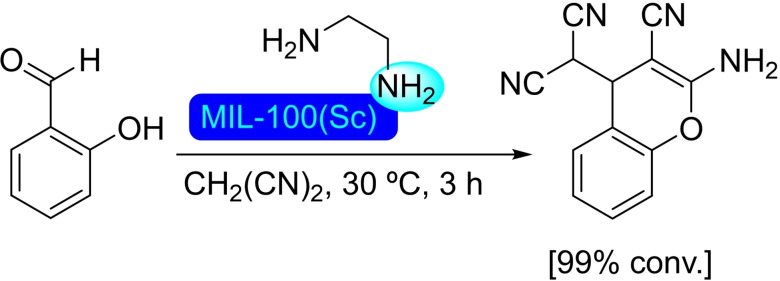
Solvent‐free synthesis of chromene catalyzed by [H_2_NCH_2_CH_2_NH_2_]@MIL‐100(Sc).

**4.5.1. Modification with amines**. The modification of MIL‐100(Cr) with ethylenediamine by SALI is analogous to that of other MOFs, in which one of the amino groups anchors to the nodes while the other is free to interact with any suitable species. This modification results in increased uptake of acidic compounds,^[161]^ as well as enhanced water adsorption capacity.^[162]^ A particularly interesting example involves the insertion (by SALI in toluene) and posterior covalent modification of ethylenediamine with paraformaldehyde and phosphorous acid to obtain [H_2_NCH_2_CH_2_N(CH_2_PO_3_H_2_)_2_]@MIL‐100(Cr). This Brønsted acidic material was effective for the multicomponent preparation of a broad variety of complex pyrimido[4,5‐*b*]quinolines.^[163]^ Similarly, ethylenediamine has been inserted in MIL‐100(Al) providing a material with both Brønsted acidic and basic sites.^[164]^ In addition, MIL‐100(Sc) has been altered with ethylenediamine and *N*,*N*’‐dimethylethylenediamine, providing catalytic materials which have been tested in the preparation of chromenes from salicylaldehyde and ethyl cyanoacetate with increased activity over the pristine structure (Scheme 50).^[165]^ In addition, aminomethanesulfonic acid has been included in the MIL‐100(Cr) attached by the amino moiety leaving the sulfonic acid group to modulate the adsorption properties of the material towards basic compounds.^[161]^

**4.5.2. Modification with alcohols**. MIL‐100(Cr) has been grafted with alcohols, such as methanol, trifluoroethanol, and hexafluoropropan‐2‐ol by the SALI. The presence of the alcohol molecules has transformed the Cr(III) Lewis acidic sites, into Brønsted acidic sites, with OH moieties.^[166]^ Similarly, ethylene glycol, diethylene glycol and triethylene glycol have been implanted in the nodes of MIL‐100(Cr), increasing the affinity of the material for water adsorption.^[162]^ On a different note, Doxorubicin has been grafted onto nanoparticles of MIL‐100(Fe), up to 9 % weight, by coordination of hydroxyl moieties of the drug with the iron present at the nodes.^[167]^

### 4.6 MIL‐101

**Scheme 51:**
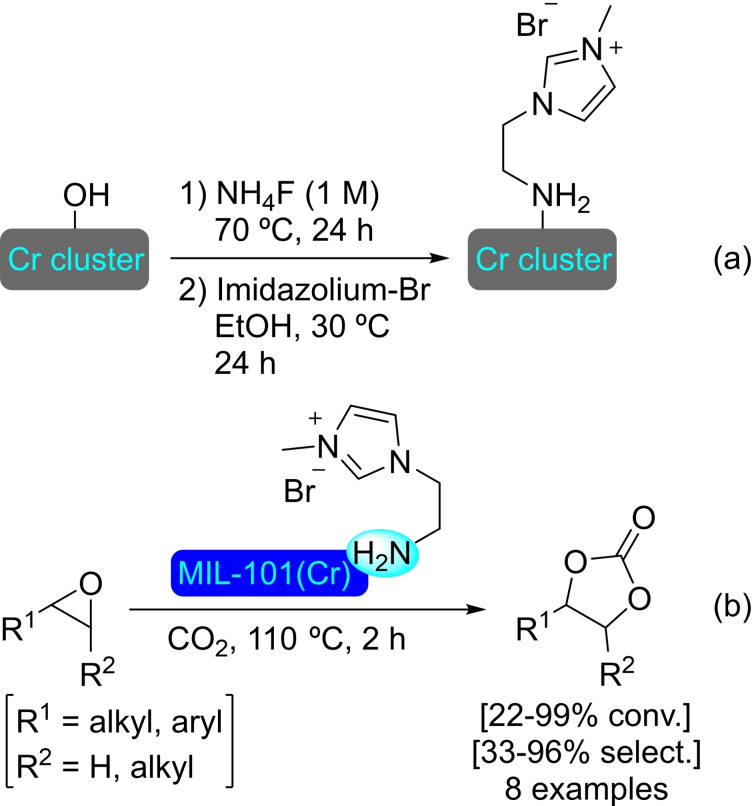
(a) Proposed model of modified MIL‐101(Cr) with an imidazolium salt, and (b) ring‐opening of epoxides with CO_2_ using functionalized MOF as catalyst.

**4.6.1. Modification with amines**. MIL‐101(Cr) exhibits chromium(III) clusters with coordinatively unsaturated sites that can act as Lewis acidic sites where amines can easily coordinate. Thus, polyamines (such as ethylenediamine,[^168^, ^169^, ^170^, ^171^] diethylenetriamine,^[172]^ and tetraethylenepentamine[^173^, ^174^]) and 1‐(2‐aminoethyl)‐3‐methylimidazolium bromide^[175]^ have been immobilized on the MOF. The amine grafting did not produce any change in the crystallinity of the material, although its surface area was reduced. The MIL‐101(Cr) altered with ethylenediamine has been described as basic adsorbent for acidic species.^[171]^ Interestingly, the presence of the amine molecules decreases the adsorption of CO on the MOF,^[170]^ which would bind directly to the node metal, but the adsorption of CO_2_,[^172^, ^174^] which can bind to the amino moieties on the grafted molecules, especially on tetraethylenepentamine, is maintained or increased. This can be exploited to facilitate the separation of these gases.^[173]^ The MIL‐101(Cr) modified with the imidazolium salt increased also the adsorption of CO_2_, which could then be reacted with epoxides to form cyclic carbonates, with the MOF itself acting as catalyst (Scheme 51).^[175]^ In addition to this, MIL‐101(Cr) grafted with amino groups have been applied as catalysts in the Knoevenagel condensation, as support for ruthenium catalytic species in the transfer hydrogenation of benzene using isopropanol,^[168]^ and as support for catalytic palladium species with application in the oxidation of styrene^[169]^ or in the Heck reaction between iodobenzene and acrylic acid. Following the same protocol, dopamine has been inserted into the cluster of MIL‐101(Cr) leaving a catechol moiety available for further modifications. Thus, vanadyl acetylacetonate, VO(acac)_2_, was reacted with this dopamine‐modified MIL‐101(Cr) forming a supported vanadium complex with relevant activity in the oxidation, using *tert*‐butylhydroperoxide, of thioanisole to its sulfoxide and sulfone derivatives. Interestingly, this material could be tuned to selectively afford the sulfoxide, while the homogeneous catalyst would yield the sulfone as the major product.^[176]^

**Scheme 52:**
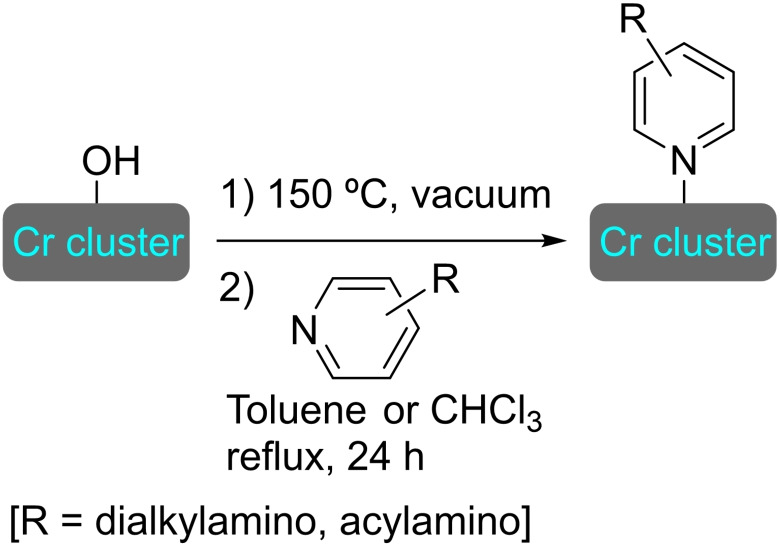
Proposed model of modified MIL‐101(Cr) with pyridine derivatives.

**Scheme 53:**
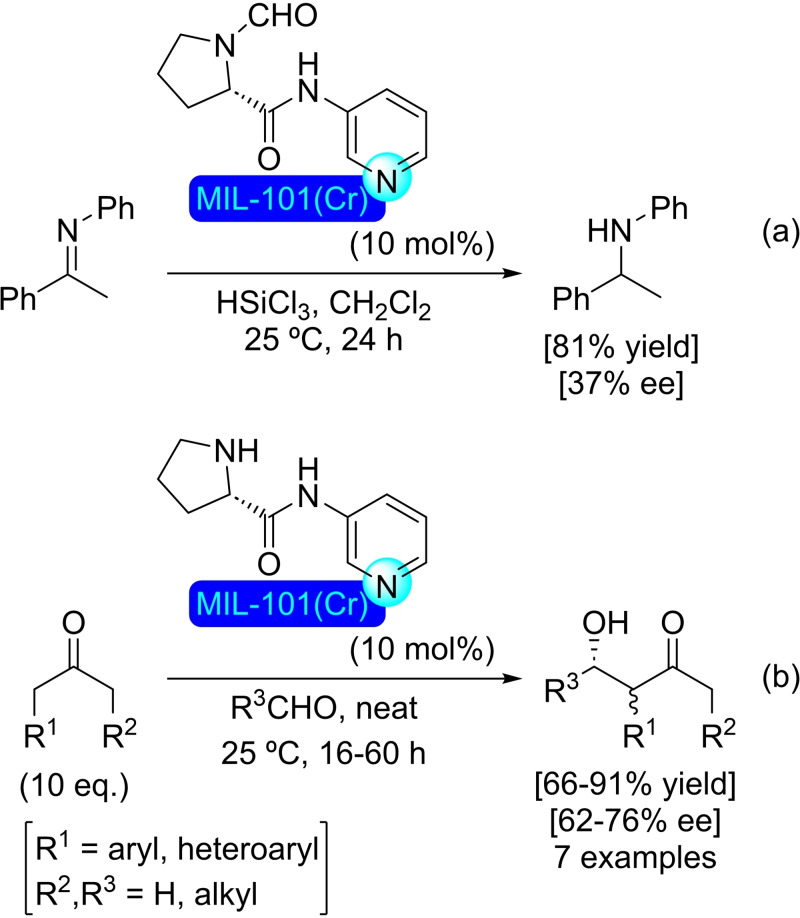
Modified MIL‐101(Cr) with chiral pyridine derivatives for asymmetric catalytic (a) reduction of imines, and (b) aldol reactions.

MIL‐101(Cr) have been also modified with aminomethanesulfonic acid,^[171]^ urea and melamine^[177]^ to generate materials with enhanced adsorption capacities due to acid‐base,^[171]^ or hydrogen‐bond interactions.^[177]^ Similarly, dialkylaminopyridines [i. e., 4‐(dimethylamino)pyridine, 4‐pyrrolidinopyridine, 4‐morpholinopyridine, and 4‐((3‐aminopropyl)methylamino)pyridine] have been included in the MIL‐101(Cr) by SALI in toluene (Scheme 52), providing catalytic materials for the hydrolytic degradation of paraoxon.^[178]^ In addition, 4’‐(4‐pyridyl)‐2,2’:6’,2’’‐terpyridine has been inserted into MIL‐101(Cr) and subsequently treated with an alkyl bromide (i. e. methyl, ethyl, butyl, and pentyl bromide) to form the corresponding pyridinium salts. The presence of these organic salts increased the carbon dioxide adsorption capacity of the MOF, which as mentioned before resulted an efficient catalyst for the reaction with epoxides to form the corresponding cyclic carbonates.^[179]^ The strategy of using pyridine units to bind to the coordination metal sites has also been employed to introduce proline derivatives in MIL‐101(Cr).[^180^, ^181^] Thus, (*S*)‐1‐formyl‐*N*‐(pyridin‐3‐yl)pyrrolidine‐2‐carboxamide has been employed to construct a MOF modified with chiral molecules (Scheme 52), with this material being employed in the asymmetric reduction of (*E*)‐*N*‐benzylidenebenzenamine using trichlorosilane, achieving good results although with low enantioselectivity (37 % ee, 81 % yield, Scheme 53).^[180]^ Similarly, (*S*)‐*N*‐(pyridine‐3‐yl)pyrrolidine‐2‐caboxyamide and (*S*)‐*N*‐(pyridine‐4‐yl)pyrrolidine‐2‐carboxyamide have been incorporated to MIL‐101(Cr) by SALI (Scheme 52), and the resulting chiral modified materials have been active catalysts in asymmetric aldol reactions with enantioselectivities up to 81 % (Scheme 53).^[181]^

**4.6.2. Modification with phosphonates**. Octadecylphosphonic acid has been grafted into the nodes of MIL‐101(Cr) to create a superhydrophobic environment. While this has no direct use as catalyst, this modification can be strategically used to stabilize sensitive species, such as copper(I) inserted within the framework. This particular material was found to be highly efficient for the selective desulfuration of fuels.^[182]^

**4.6.3. Modification with halides**. The presence of open metal sites in the clusters allows the coordination of anions. Thus, treatment of MIL‐101(Cr) with diluted solutions of ammonium chloride and fluoride resulted in the incorporation of these halides to the materials, resulting in the modulation of the Lewis acidity of the metal centers.^[183]^

### 4.7 PCN‐222 and PCN‐224

The combination of zirconium clusters with 5,10,15,20‐tetrakis(4‐carboxyphenyl)porphyrin resulted in the formation of metal‐organic frameworks with several different topologies, such as PCN‐222 and PCN‐224. PCN‐222, also known as MOF‐545 and MMPF‐6, presents a hexagonal morphology with eight linkers per node, whereas PCN‐224 has cubic structure with twelve linkers per node.^[184]^

**Scheme 54:**
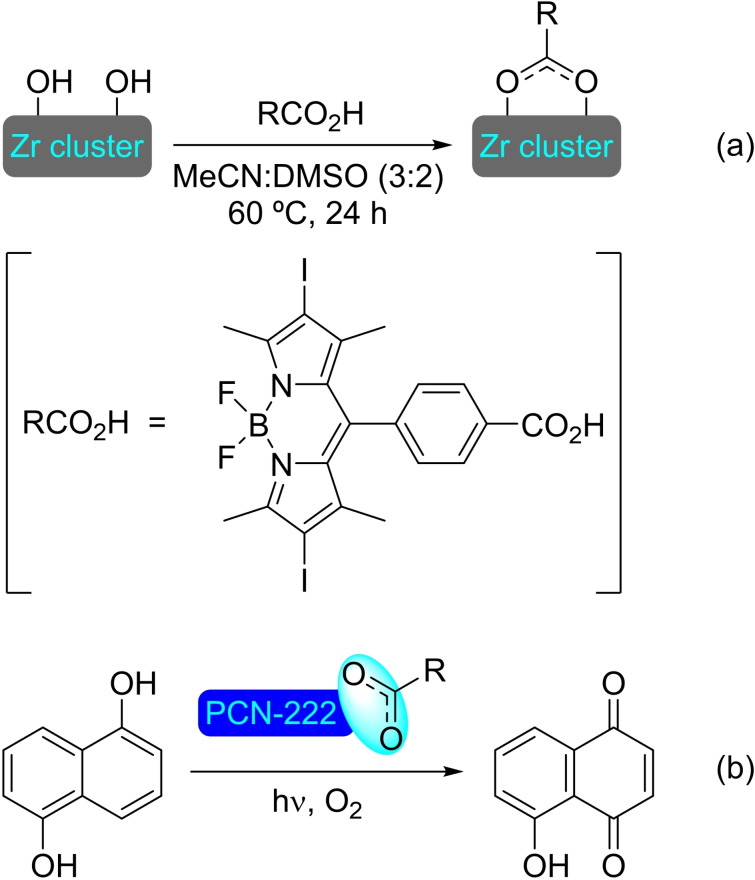
(a) Proposed model of modified PCN‐222 with a BODIPY, and (b) photooxidation of dihidroxynaphthalene modified MOF as catalyst.

**4.7.1. Modification with carboxylates**. In the same direction as other carboxylate‐linker MOFs, molecules containing carboxylic acids can bind to the nodes of PCN‐222. This way, myristic acid has been inserted into the nodes of PCN‐222 to modulate channel dimensions and pore size distribution, with the altered material being employed as a component in polyamide membranes to increase salt rejection while maintaining water permeability through the membranes.^[185]^ Moreover, mercaptoacetic acid was inserted into the MOF, by microwave assisted SALI, to improve the selective adsorption of copper(II) cations in the presence of other metal cations in wastewater.^[186]^ In addition, the molecule 4,4‐difluoro‐8‐(4’‐carboxyphenyl)‐2,6‐diiodo‐1,3,5,7‐tetramethyl‐4‐bora‐3a,4a‐diaza‐*s*‐indacene has been incorporated to PCN‐222 using the SALI approach (Scheme 54). The inclusion of this BODIPY compound, which is a photosensitizer as tested in the photooxidation of dihydroxynaphthalene (Scheme 54), resulted in a material with a higher singlet‐oxygen quantum yield that has been assayed as cytotoxic agent against cancer cells, targeting application in PDT.^[187]^ In this sense, a PCN‐224 modified by insertion of maltotrionate has been prepared and tested for cancer treatment by PDT.^[188]^ The use of porphyrin linkers with iron centers coordinated inside provided the corresponding PCN‐222(Fe). This material has been post‐synthetically modified by treatment with a solution of ferrocenecarboxylic acid, resulting in a material integrating multiple redox centers with potential applications in synergistic redox catalysis.^[189]^

**Scheme 55:**
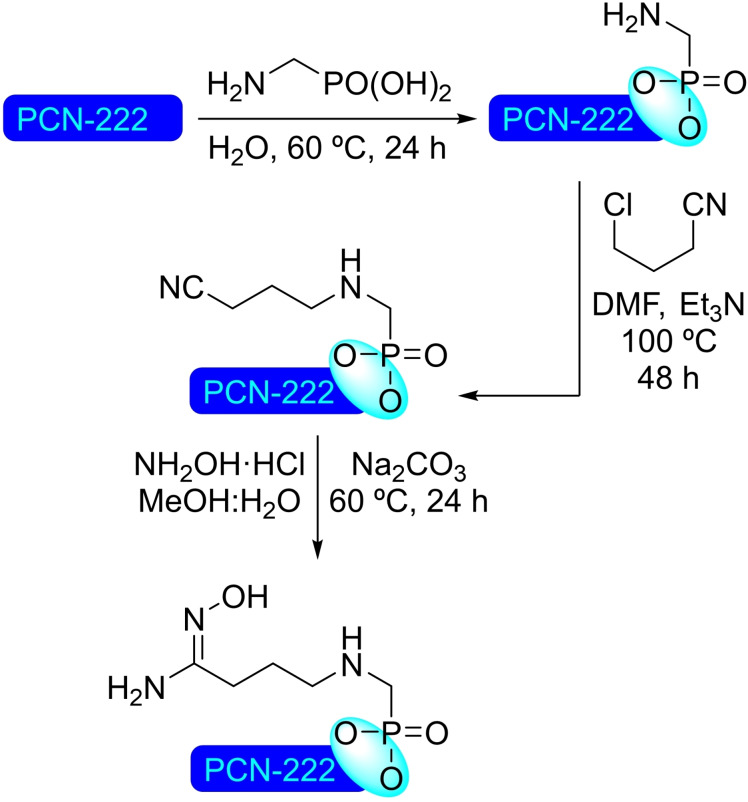
Proposed modification of PCN‐222 by SALI with aminomethylphosphonic acid, 4‐chlorobutyronitrile and hydroxylamine.

**4.7.2. Modification with phosphinates and phosphonates**. Diphenylphosphinic acid has been inserted into the nodes of PCN‐222 by SALI in different amounts up to a ratio of 20 units per node. The presence of the phosphinate resulted in no distortion of the MOF structure, although it significantly increased its hydrophobicity. After complete water removal, the functionalized material did show 4‐fold increased the singlet‐oxygen quantum yield compared to the unmodified MOF.^[190]^ On a different note, PCN‐222 has been treated with solutions of aminomethylphosphonic acid, 4‐chlorobutyronitrile, and hydroxylamine, giving an amidoxime‐functionalized material (Scheme 55), with outstanding selectivity in the absorption of uranium(VI) ions.^[191]^

**4.7.3. Modification with sulfonates**. The presence of OH groups in the zirconium clusters and the large pores of PCN‐222 have been used to graft benzene‐1,3,5‐trisulfonic acid, by SALI. The inclusion of these molecules resulted in a dramatic reduction of the pore volume, but vastly increased water affinity even at low relative humidity.^[192]^

### 4.8 HKUST‐1

Benzene‐1,3,5‐tricarboxylic acid as linker in combination with copper(II) cations gives rise to the MOF described as HKUST‐1, which is often referred to as Cu(BTC) as well.^[193]^ The linkers connect copper dimers, which has free coordination positions saturated by the solvent used in the synthesis (usually water). Activation of the material by vacuum heat treatment releases these positions where other species can be readily inserted for the post‐functionalization of the MOF. Contrary to zirconium and hafnium MOFs, nitrogenated moieties are most suitable for insertion, although care must be taken not to completely displace the constituent linkers, due to the higher affinity of copper for nitrogen rather than oxygen.

**Scheme 56:**
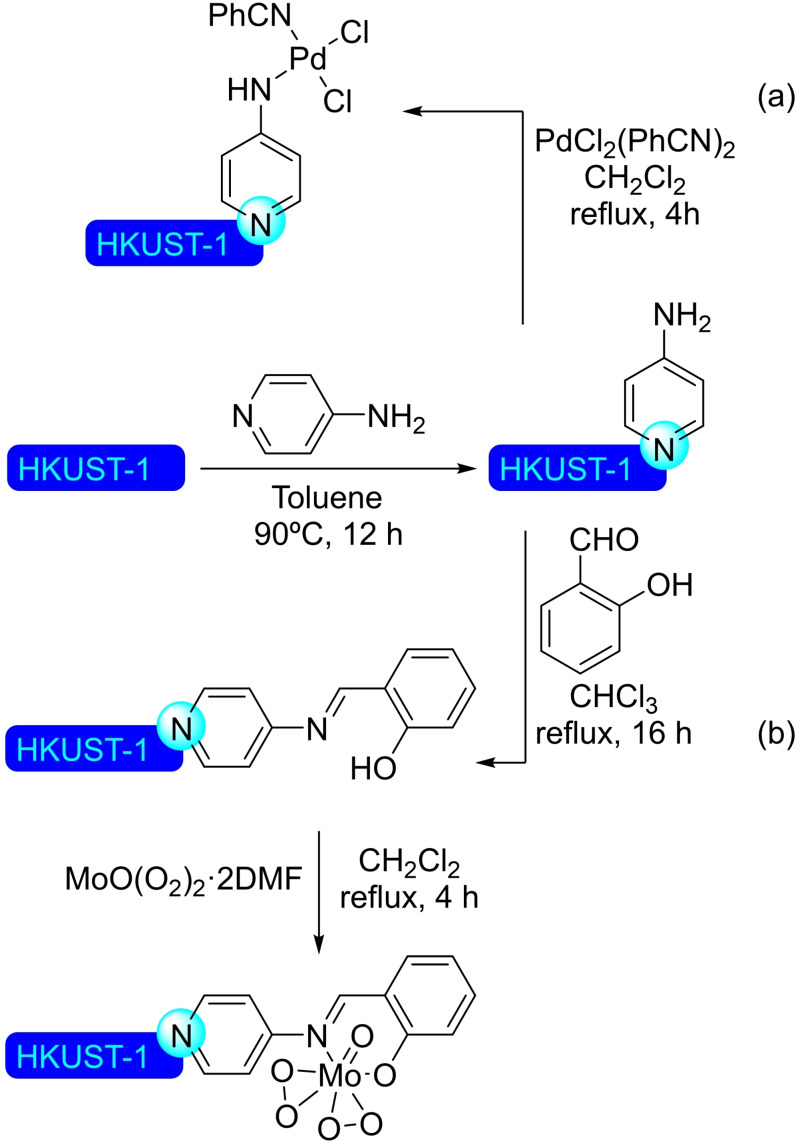
Proposed modification of HKUST‐1 by SALI with pyridines to form grafted catalysts to support (a) a palladium complex, and (b) a molybdenum complex.

**Scheme 57:**
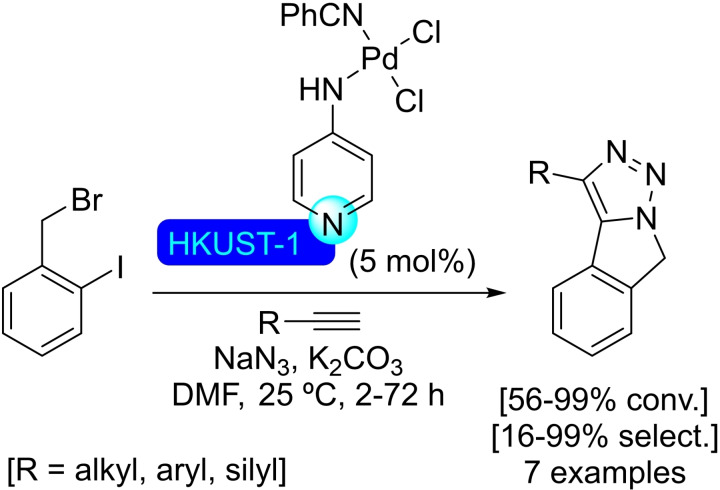
Domino Sonogashira coupling/click cyclization for the synthesis of triazolo[5,1‐*a*]isoindoles catalyzed by [Pd‐aminopyridine]@HKUST‐1.

**Scheme 58:**
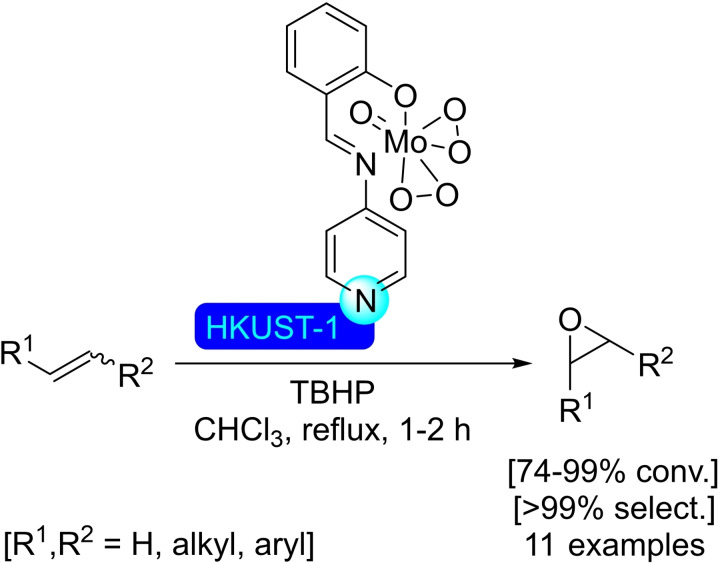
Olefin epoxidation catalyzed by [Mo‐salen‐pyridine]@HKUST‐1.

**4.8.1. Modification with amines**. Since the first synthesis of HKUST‐1, pyridine derivatives have been considered as prime candidates to be inserted into the dimeric metal centers.^[193]^ In general, ligand insertion is carried out by the SALI approach, prior activation of the material, although the insertion of 4‐(methylamino)pyridine by phase‐vapor diffusion at high temperature has also been described.^[194]^ Pyridine derivatives, such as 4‐aminopyridine and 2‐(*tert*‐butyl)‐4‐methyl‐6‐[(pyridine‐4‐ylimino)methyl]phenol, have been grafted on the copper nodes forming materials with ligands to support metal complexes enabling different types of transformations (Scheme 56).[^195^, ^196^, ^197^] Palladium(II) complexes have been supported on these modified HKUST‐1, resulting in a bifunctional catalyst able to perform tandem Sonagashira‐click reactions (Scheme 57). The catalysts resulted very active and selective in these transformations.^[195]^ In addition, molybdenum‐aminopyridine complexes have been set up at the nodes HKUST‐1 resulting in catalytic materials for the epoxidations of olefins, using *tert*‐butylhydroperoxide as oxidant, with high conversions and excellent selectivities (Scheme 58).[^196^, ^197^] The amino group in the ligand has also served to immobilize the complex bis(acetylacetonato)dioxomolybdenum(VI) generating an active oxidation catalyst;^[196]^ besides, a second version where the amino group has been reacted with salicylaldehyde to form a salen‐type ligand which has allowed the immobilization of oxodiperoxomolybdenum(VI), thus demonstrating possible approaches in the formation of grafted catalysts.^[197]^

Glycine,^[198]^ 1‐(2‐aminoethyl)‐3‐methylimidazolium hydroxide,^[199]^ ethylenediamine, and diethylenetriamine^[165]^ have been inserted in the nodes of the MOF, maintaining the crystallinity of the material and, in the case of the glycine, increasing its stability.^[198]^ The HKUST‐1 functionalized with the imidazolium salt resulted to be an active catalyst in the Knoevenagel condensation between malononitrile and different benzaldehydes, interestingly exhibiting size‐selective behavior.^[199]^ The materials altered with ethylenediamine and diethylenetriamine have been successfully tested in the reaction between salicylaldehyde and ethyl cyanoacetate to afford chromenes.^[165]^

**Scheme 59:**
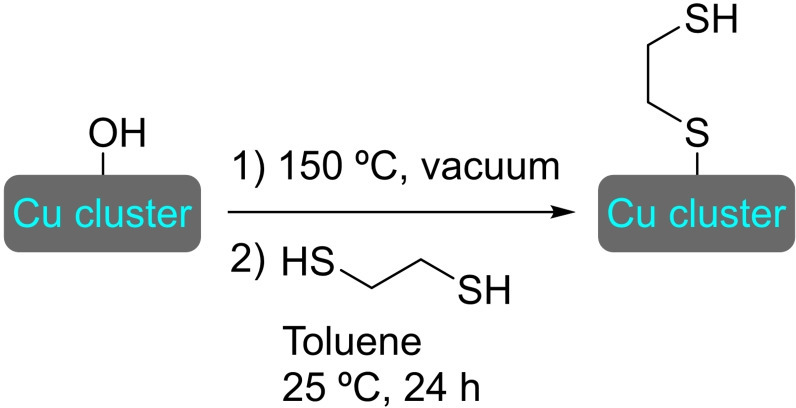
Proposed modification of HKUST‐1 by SALI with ethane‐1,2‐dithiol.

**4.8.2. Modification with thiols**. HKUST‐1 has been modified with dithioglycol, by insertion of one of the thiol groups into the nodes (Scheme 59). The modified material has been tested in the selective adsorption of mercury(II) ions from water solutions, exploring its application for the selective removal of heavy metal ions from wastewater.^[200]^

### 4.9 Cu‐BTTri Modified with Amines

This MOF can be synthetized from a copper(II) salt and 1,3,5‐tris(1*H*‐1,2,3‐triazol‐5‐yl)benzene as linker, the structure being formed by square chloride‐centered clusters (Cu_4_Cl)^+7^ linked by triangular (BTTri)^−3^ with a solvent molecule (usually DMF) coordinated to each copper(II) species. The elimination of the solvent molecule at the metal center allows the insertion of other types of ligands. Thus, ethylenediamine^[201]^ and N,N’‐dimethylethylenediamine^[202]^ have been implemented, by SALI, to Cu‐BTTri resulting in materials with high adsorption for carbon dioxide.

### 4.10 Cu‐BDC Modified with Amines

**Scheme 60:**
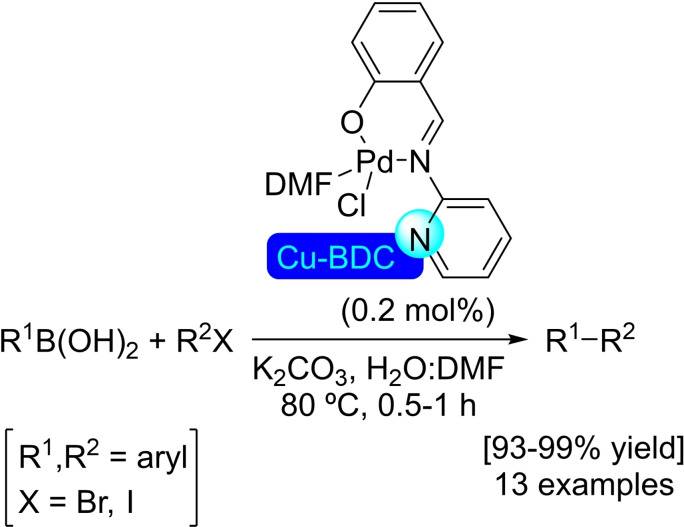
Suzuki‐Miyaura coupling for the synthesis of biaryl compounds catalyzed by [Pd‐salen‐pyridine]@Cu‐BDC.

Copper(II) cations can be connected with 1,4‐benzenedicarboxylic acid to form a MOF with similar coordinative sites in the nodes to HKUST‐1.^[203]^ Thus, pyridine ligands can be inserted into the material by SALI methodology, which have been proven by the addition of a pyridyl‐salicylimine ligand. The ligand formed by the coupling of 2‐aminopyridine and salicylaldehyde has been employed to support palladium(II) species, which have been tested successfully in the Suzuki cross‐coupling reaction between arylboronic acids and aryl iodides or bromides (Scheme 60).^[204]^

### 4.11 Cu‐bcmim Modified with Halides

**Scheme 61:**
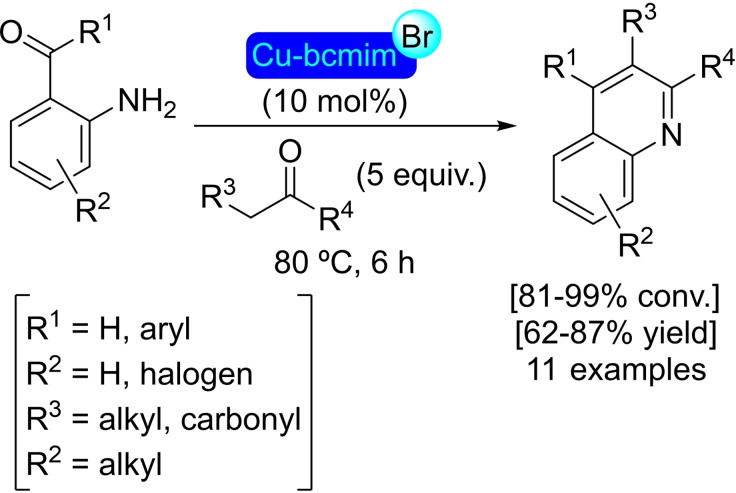
Synthesis of quinolines catalyzed by [Br]@Cu‐bcmim.

The combination of copper(II) acetate and 1,3‐bis(carboxymethyl)imidazole (bcmim) gives rise to Cu(bcmim), in which the copper cations are bridged by bcmim units with a square‐planar disposition, resulting in a laminar structure.^[205]^ MOFs containing this type of linker are well suited for post‐modification, due to the loose coordination and flexibility this moiety provides, facilitating the installation of different molecules at the node. In this sense, Cu(bcmim) was modified with bromide and chloride by SALI in methanol with the corresponding hydrohalic acid solution, observing the incorporation of 0.9 chloride and 0.7 bromide anions per node, evidenced by a significant change in color of the material. The modified material experienced a significant enhancement in catalytic activity, presenting much increased Lewis acidity towards epoxide ring opening and Friedländer reactions (Scheme 61), as well as higher activity in the oxidative coupling of formamides and carboxylic acids compared to the pristine material.^[206]^

**5. Summary and Outlook**

The post‐synthetic modification of metal‐organic frameworks is a strategy to broaden the range of applications of these materials. Among all available strategies, the functionalization of metal nodes with other metal species or with molecules with chelating groups that binds to the node offers interesting possibilities while retaining a high degree of operational simplicity. Most metal‐organic frameworks that have been used for modification are based on transition metals such as zirconium, titanium or hafnium due to their higher stability, although there are examples based on other metals such as iron, copper, manganese or chromium. The modification with metals has been carried out using highly active metal reagents by techniques such as ALD, or solvothermal immersion. The inserted metal at the node may remain in cationic form (stabilized by the node environment and by other ligands), be part of a subcluster built on top of the node, which also acts as template, or may be reduced to give rise to metal(0) species in the form of isolated atoms or nanoparticles, with the deposition of two metal species also having been described. The presence of other metals in the MOF nodes modifies the physicochemical properties of the MOFs giving rise to heterogeneous catalysts with different applications, often targeting modulation of the catalytic properties of the constituent metal (e. g. Lewis acidity) or more complex synergistic polymetallic catalysis. Albeit mostly as proofs of concept to showcase the potential of modified MOFs, many different catalytic processes have been tested. Thus, oxidation reactions (such as epoxidation of alkenes, oxidation of methane and other alkanes, oxidation and photooxidation of benzyl alcohol derivatives, water oxidation, dehydrogenation of cyclohexene to benzene), reduction reactions (such as reduction of nitro compounds, nitriles and isocyanides, reduction of alkenes, Meerwein‐Ponndorf‐Verley reduction, hydrogenation of arenes and heteroarenes), metathesis of simple alkenes, olefin polymerization, hydroformylation, intramolecular hydroamination, isomerization of glucose have been tested with metal‐modified metal‐organic frameworks, showing increased activities and selectivities over the pristine materials or analogous systems based on the newly inserted metal. Table 1 outlines the modifications of nodes with metallic species and their respective catalytic applications. On the other hand, the implantation of organic molecules in the nodes is another obvious modification path due to the presence of functional groups that can be attached to coordinative sites in the nodes. As anchoring groups, carboxylates, phosphates, phosphonates, sulfates, sulfamates, persulfates, alcohols and amines have all been used. The main methodology used to carry out the functionalization with organic molecules is SALI, due to its effectiveness and simplicity. The presence of the organic molecules may alter or add physical and/or chemical properties, such as Lewis and Brønsted acidity, electronic conductivity, electroactivity, electrochemiluminescence, visible‐light absorption or redox properties. In addition, these molecules can be further altered by different transformations in heterogeneous phase, giving rise to a potentially infinite range of modifications. Metal‐organic frameworks modified with different organic molecules have proven to be useful for the selective adsorption of gases (i. e. CO_2_ or SO_2_), bioactive compounds, chemical warfare agents and metal cations [i. e. Cu(II) or U(VI)] from wastewater. Regarding catalysis, many reactions, such as ring opening of epoxides, dimerization of isobutene, transfer hydrogenation with isopropanol, epoxidation of olefins, isomerization of glucose to 5‐hydroxymethylfurfural, photocatalytic oxidations, Knoevenagel condensation, and synthesis of 2‐arylbenzoxazoles and chromenes have been successfully carried out with activities and selectivities much higher than those of the unmodified materials or the corresponding homogeneous phase catalysts. The inclusion of chiral molecules has allowed the catalytic asymmetric reductions and asymmetric aldol reaction, although more work is required to fine tune the mechanisms of asymmetric induction within the tight environment of the MOF pores. Metal complexes with anchoring groups have been implemented in the material providing catalytic materials for transformations such as ethylene hydrogenation with an iridium complex, dimerization of ethylene with a nickel complex, cycloaddition reactions with a copper complex, oxidation of thioanisole with a vanadium complex, epoxidation of alkenes with a molybdenum complex, and Suzuki cross‐coupling reaction, Sonagashira reaction and oxidative Heck reaction with palladium complexes. Inserting complexes in metal‐organic frameworks is also beneficial from a stability standpoint, as the node is highly protected from the outside environment, which drastically increases the lifespan of the catalysts and enables the use of highly sensitive complexes. Table 2 summarizes node modifications with organic molecules, along with their corresponding catalytic applications.

**Table 1:** Post‐synthetic modifications of nodes with metallic species and their catalytic uses.

Entry	MOF	Metal	Modification procedure	Reaction	Ref.
1	Zr‐NU‐1000	Al	ALD/SIM with AlMe_3_	Knoevenagel condensation	32
2	Zr‐NU‐1000	Al	ALD with i‐PrOAlMe_2_	Ethanol dehydration	33
3	Zr‐NU‐1000	Fe	SIM with Fe(NO_3_)_3_ or FeCl_2_	Oxidation of cyclohexene	35
4	Zr‐NU‐1000	Co	ALD with Co(MeC(NiPr)_2_)_2_ and H_2_S	Reduction of nitro compounds and dehydrogenation of propane	37,38
5	Zr‐NU‐1000	Ni	ALD with Ni(MeC(N*t*‐Bu)_2_)_2_	Ethylene oligomerization	41
6	Zr‐NU‐1000	Ni	SIM with Ni(OAc)_2_ or Ni(MeC(N*t*‐Bu)_2_)_2_ and H_2_O	Flow ethylene hydrogenation	42,43
7	Zr‐NU‐1000	Ni	ALD with Ni(MeC(N*t*‐Bu)_2_)_2_ and H_2_S	Photocatalytic hydrogen evolution	44
8	Zr‐NU‐1000	Cu	ALD with Cu(dmap)_2_ and H_2_O	Oxidation of methane	45
9	Zr‐NU‐1000	Zn	ALD/SIM with ZnEt_2_	Knoevenagel condensation	32
10	Zr‐NU‐1000	Nb	ALD or condensed phase grafting with TBTDEN and H_2_O	Oxidation of cycloalkanes with H_2_O_2_	49
11	Zr‐NU‐1000	Mo	SIM with TBTDEM and H_2_O	Epoxidation of alkenes	50
12	Zr‐NU‐1000	Mo	SIM with TBTDEM and H_2_S	Electrocatalytic water splitting	51
13	Zr‐NU‐1000	Mo	ALD with Mo(CO)_6_ and H_2_S	Desulfurization of dibenzothiophene	52
14	Zr‐NU‐1000	W	Conventional heating under vacuum with W(≡C*t*‐Bu)(CH_2_ *t*‐Bu)_3_	Metathesis of alkenes	53
15	Zr‐NU‐1000	Re	ALD with MeReO_3_	Flow hydrogenation of alkenes and olefin metathesis	54,55
16	Zr‐NU‐1000	Ir	SIM with Ir(CO)_2_ or Ir(C_2_H_4_)_2_	Flow hydrogenation of alkenes	56
17	Zr‐NU‐1000	Pt	ALD with MeCpPtMe_3_	Hydrogenation of alkenes	57
18	Zr‐NU‐1000	U	SIM with UO_2_(OAc)_2_	Photooxidation of benzyl alcohols	58
19	Zr‐NU‐1000	Al, Co	SIM with (py_3_tren)AlCo	Oxidation of benzyl alcohols	59
20	Zr‐NU‐1000	Ni, Zn, Al, Ti or Mo and Co	SIM with the first metal precursor, then ALD with Co(MeC(NiPr)_2_)_2_	Oxidation of propane to propene	60
21	Zr‐NU‐1000	Zn, Co	ALD with CpCo(CO)_2_ then ZnEt_2_	Reduction of propyne	61
22	Hf‐NU‐1000	Zr	SIM with Zr(Bn)_4_	Olefin polymerization	63
23	Zr and Hf‐NU‐1200	Ni	SIM with Ni(OAc)_2_	Flow hydrogenation of ethylene	42
24	Zr‐NU‐1200	Mo	SIM with MoO_2_(acac)	Oxidation of benzyl alcohols with oxygen	65
25	Zr‐UiO‐66 and Zr‐UiO‐66‐NH_2_	Al	SIM with AlMe_3_ and iPrOH	Meerwin‐Ponndorf‐Verley reduction of cyclohexanone	67
26	Zr‐UiO‐66	Ti	SIM with TiO(acac)_2_	Oxidation of cyclohexene with H_2_O_2_	69
27	Zr‐UiO‐66	V	SIM with VO(acac)_2_	Dehydrogenation of cyclohexene	70
28	Zr‐UiO‐66	Fe	SIM with FeCl_2_	Oxidation of methane with H_2_O_2_	71
29	Zr‐UiO‐66‐NH_2_	Co	SIM with CoCl_2_	Boosting luminol‐H_2_O_2_ chemoluminiscence reaction	72
30	Zr‐UiO‐66	Ni	ALD with Ni(MeC(N(iBu))_2_)_2_	Flow hydrogenation of ethylene	73
31	Zr‐UiO‐66‐NH_2_	Ni	MW‐SIM with NiCl_2_ and HCSNH_2_	Photocatalytic water splitting	74
32	Zr‐UiO‐66	Nb	SIM with NbCl_5_	Isomerization of glucose to fructose	75
33	Zr‐UiO‐66	Mo	SIM with H_3_PMo_12_O_40_	Oxidative desulfurization	76
34	Zr‐UiO‐66	Rh	SIM with Rh(acac)(C_2_H_4_)_2_	Hydroformylation of butene	77
35	Zr‐UiO‐66	Ir	SIM with Ir(CO)_2_ or Ir(C_2_H_4_)_2_	Flow hydrogenation of ethylene	56
36	Ce‐UiO‐66	Cu	SIM with Cu(OAc)_2_	Ammonia to nitrate conversion	80
37	Zr‐UiO‐67	Al	SIM with AlMe_3_ and treatment with iPrOH	Meerwin‐Ponndorf‐Verley reaction of cyclohexanol	67
38	Zr‐UiO‐68	Co	SIM with CoCl_2_ and reduction with NaEt_3_BH	Hydrogenation and hydroboration of olefins	82
39	Zr‐UiO‐68	Fe	SIM with FeBr_2_	Amination of C−H bonds with aniline	82
40	Zr‐UiO‐69	Mg	SIM with Me_2_Mg	Hydroboration of carbonyls and imines, hydroamination of 4‐pentenylamines	83
41	Zr(mtbc)	Co	SIM with CoCl_2_ and reduction with NaEt_3_BH	Hydrogenation of olefins, carbonyls, and imines	85
42	NPF‐520	Fe	Treatment with (Me_3_Si)CH_2_Li and SIM with FeCl_3_	Photocatalytic oxidation of toluene	86
43	Zn‐MOF‐5	Rh	SIM with Rh(acac)(C_2_H_4_)_2_	Hydroformylation of butene	77
44	Zr‐MOF‐808	Fe	SIM with Fe_2_(acac)_3_	Oxidation of benzyl alcohols with TBHP	88
45	Hf‐MOF‐808	Pd	SIM with Pd(OAc)_2_	Heck reaction with 2‐phenylphenol and ethyl acrylate	89
46	Zr‐HUST‐1	Ni	SIM with NiX_2_ (X=Cl, Br, NO_3_, OAc) or NiC_2_O_4_	Dimerization of ethylene	90
47	Zr‐PCN‐222(Fe)	Fe	SIM with FeCl_2_	Aerobic oxidation/cyclization of benzyl alcohols and 2‐aminobenzamides to quinazolin‐4‐ones	91
48	Al‐DUT‐5	Al	SIM with AlMe_3_ and treatment with iPrOH	Meerwin‐Ponndorf‐Verley reaction of cyclohexanol	67
49	Fe‐MIL‐101	Cu	SIM with CuCl	Detoxification of organic pollutants with KHSO_5_	94
50	Fe‐MIL‐101	Rh	SIM with Rh(acac)(C_2_H_4_)_2_	Hydroformylation of butene	77
51	Ti‐MIL‐125	Co	SIM with CoCl_2_ and reduction with NaEt_3_BH	Hydrogenation of arenes and heteroarenes	96
52	Ti‐MIL‐125‐NH_2_	Cu	SIM with [(CH_3_CN)_4_Cu]BF_4_	Reduction of CO_2_ to ethylene	97

**Table 2:** Post‐synthetic modifications of nodes with organic species and their catalytic uses.

Entry	MOF	Grafted compound(s)	Modification procedure	Reaction	Ref.
1	Zr‐NU‐1000	Carboxy(alkylpyridinium) salts	SALI with 2‐, 3‐, and 4‐carboxypyridines followed by alkylation with alkyl halides	Capture and reaction of CO_2_ with epoxides	104
2	Zr‐NU‐1000	PCBA	SALI with PCBA	Photocatalytic oxidation of sulfur mustard	109
3	Zr‐NU‐1000	BODIPY‐carboxylates	SALI with BODIPY‐carboxylates	Photooxidation of 1,5‐hydroxynaphthalene to juglone	110
4	Zr‐NU‐1000	5‐(Carboxymethoxy)‐1,3‐bis(di‐*tert*‐butylphosphite)‐benzene iridium(III) hydride	SALI with the complex	Hydrogenation of alkenes	117
5	Zr‐NU‐1000	[Rh(Cp*)(5‐carboxy‐bpy)Cl]^+^	SALI with the complex	Electrocatalytic reduction of NAD to NADH	118
6	Zr‐NU‐1000	Phosphate	Treatment with aqueous phosphoric acid	Isomerization‐dehydration of glucose to 5‐HMF	119
7	Zr‐NU‐ 1000	Bipyridine‐Ni(II) complex	SALI with 5‐methylphosphonate‐2,2’‐bipyridine, then with NiCl_2_ and activation with Et_2_AlCl	Dimerization of ethylene to butene	121
8	Zr‐UiO‐66	d‐gluconate	SALI with d‐gluconic acid	Cycloaddition of CO_2_ to epoxides	122
9	Zr‐UiO‐66(Fe)	Octadecylphosphonate	SALI with octadecylphosphonic acid	Reduction of O_2_ to H_2_O_2_	130
10	Zr‐UiO‐66/NH_2_ (3 : 1)	*tert*‐Butoxide	SALI with LiO*t*Bu	Hydrolysis of soman and mustard gas	132
11	Zr‐MOF‐808	EDTA−Pd complex	SALI with Na_2_EDTA followed by treatment with Pd(II) salts	Suzuki coupling of iodoarenes and boronic acids	146
12	Zr‐MOF‐808	Cu(I)‐catechol complex	SALI with 2,3‐ and 3,4‐dihydroxybenzoic acid followed by treatment with CuBr	Alkyne‐azide cycloaddition	151
13	Hf‐MOF‐808	Pd(II)‐phosphate or sulfate complexes	SALI with H_3_PO_4_ or H_2_SO_4_ followed by treatment with Pd(II) salts	Oxidative Heck reaction	89
14	Zr‐MOF‐808	Sulfate	SALI with H_2_SO_4_	Selective dimerization of isobutene	155
15	Hf‐MOF‐808	Sulfate	SALI with H_2_SO_4_	Synthesis of 2‐arylbenzoxazoles	157
16	Zr‐MOF‐808	Methoxide	Prolonged heating in MeOH	Transfer hydrogenation of furfural to furfuryl alcohol	158
17	Cr‐MIL‐100	H_2_NCH_2_CH_2_N(CH_2_PO_3_H_2_)_2_	SALI with ethylenediamine followed by treatment with paraformaldehyde and phosphorous acid	Synthesis of pyrimido[4,5‐*b*]quinolines by multicomponent reaction.	163
18	Sc‐MIL‐100	Ethylenediamine and *N*,*N*‐dimethylethylenediamine	SALI with the corresponding amine	Synthesis of chromenes from salicylaldehydes and ethyl cyanoacetate	165
19	Cr‐MIL‐101	1‐(2‐Aminoethyl)‐3‐methylimidazolium bromide	SALI with 1‐(2‐aminoethyl)‐3‐methylimidazolium bromide	Cycloaddition of CO_2_ to epoxides	175
20	Cr‐MIL‐101	Ethylenediamine, diethylenetriamine and their corresponding Pd(II) complexes	SALI with the amines, then treatment with PdCl_2_ for the Pd‐loaded MOFs	Knoevenagel condensation of benzaldehyde and ethyl cyanoacetate (free amines), Heck reaction (Pd‐loaded)	170
21	Cr‐MIL‐101	Ethylenediamine, diethylenetriamine and their corresponding Pd(II) complexes	SALI with the amines, then treatment with PdCl_2_ for the Pd‐loaded MOFs	Knoevenagel condensation of benzaldehyde and malononitrile (free amines), Heck reaction (Pd‐complexes)	172
22	Cr‐MIL‐101	Ethylenediamine‐[Ru_3_(μ_3_‐O)(μ‐OAc)_6_(H_2_O)_3_]OAc) complex	SALI with ethylenediamine followed by treatment with a solution of the Ru(III) complex	Catalytic transfer hydrogenation of benzene	168
23	Cr‐MIL‐101	Ethylenediamine‐Pd(II) complex	SALI with ethylenediamine followed by treatment with Pd(OAc)_2_	Oxidation of styrene, hydrogenation of phenylacetylene, Heck reaction	169
24	Cr‐MIL‐101	V(IV)‐dopamine complex	SALI with dopamine followed by protection with tert‐butyldimethylsilyl and metalation with VO(acac)_2_	Oxidation of thioanisole with TBHP	176
25	Cr‐MIL‐101	Dialkylaminopyridines	SALI with the corresponding dialkylaminopyridine	Hydrolysis of paraoxon	178
26	Cr‐MIL‐101	Alkyl 4’‐(4‐pyridyl)‐2,2’:6’,2’’‐terpyridinium salts	SALI with 4’‐(4‐pyridyl)‐2,2’:6’,2’’‐terpyridine followed by alkylation	Cycloaddition of CO_2_ to epoxides	179
27	Cr‐MIL‐101	(*S*)‐1‐Formyl‐*N*‐(pyridin‐3‐yl)pyrrolidine‐2‐carboxamide	SALI with the pyridine‐grafted proline derivative	Enantioselective reduction of benzylidenebenzeneamide with trichlorosilane	180
28	Cr‐MIL‐101	(*S*)‐*N*‐(Pyridine‐3‐yl)pyrrolidine‐2‐caboxyamide and its pyridine‐4‐yl analogue	SALI with the pyridine‐grafted proline derivative	Enantioselective aldol reactions	181
29	Cu‐HKUST‐1	Aminopyridine‐Pd(II) complexes	SALI with the aminopyridine followed by treatment with PdCl_2_(PhCN)_2_	Sequential Sonogashira and alkyne‐azide cycloaddition	195
30	Cu‐HKUST‐1	Aminopyridine‐Mo(VI)	SALI with 4‐aminopyridine followed by treatment with MoO_2_(acac)_2_	Epoxidation of olefins with TBHP	196
31	Cu‐HKUST‐1	Aminopyridine salen‐Mo(VI) complex	SALI with 4‐aminopyridine followed by treatment with salicylaldehyde, then with MoO_2_(acac)_2_	Epoxidation of olefins with TBHP	197
32	Cu‐HKUST‐1	1‐(2‐aminoethyl)‐3‐methylimidazolium hydroxide	SALI with the imidazolium salt	Knoevenagel condensation of benzaldehydes and malononitrile	199
33	Cu‐HKUST‐1	Ethylenediamine and *N*,*N*‐dimethylethylenediamine	SALI with the corresponding amine	Synthesis of chromenes from salicylaldehydes and ethyl cyanoacetate	165
34	Cu‐BDC	Aminopyridine salen‐Pd(II) complex	SALI with the aminopyridine salen followed by treatment with PdCl_2_	Suzuki coupling of arylboronic acids and aryl halides	204
35	Cu‐bcmim	Bromide	SALI with HBr	Friedländer reaction	206

In general, post‐synthetic methodologies for inserting both metals and organic molecules into the nodes of metal‐organic frameworks are well defined, although novel techniques are constantly in development. Although as mentioned, most studies carried out to date are only proofs of concept, the usefulness of modified MOFs to obtain highly task specific materials for almost any application has been more than proven, offering just a sample of the wide possibilities of these outstanding materials, which will definitely be expanded in the coming years. Once challenges related to the highly solvent and energy intensive syntheses as well as their limited stability compared to conventional supports (especially in the humid and harsh industrial environment) are adressed, metal‐organic frameworks are prompted to become the catalytic materials of the future.

## Conflict of Interests

The authors declare no conflict of interest.

## Biographical Information

*Mario Martos (Ibi, Spain, 1996) obtained his PhD in Organic Synthesis under the supervision of Prof. Isidro M. Pastor at the University of Alicante (Spain) in 2023. After a brief postdoctoral stay at the same group, he pursued further studies at the MATCAT group of the same university, supervised by Assoc. Prof. Bosque and Prof. Gonzalez‐Gomez, working in photoredox catalysis. Since September 2024, he holds a Marie Skłodowska‐Curie Actions postdoctoral fellowship in the group of Prof. Henrik Sundén at the University of Gothenburg (Sweden). He is currently working towards the development of active materials based on oxotriphenylhexanoate (OTHO) hydrogels*.

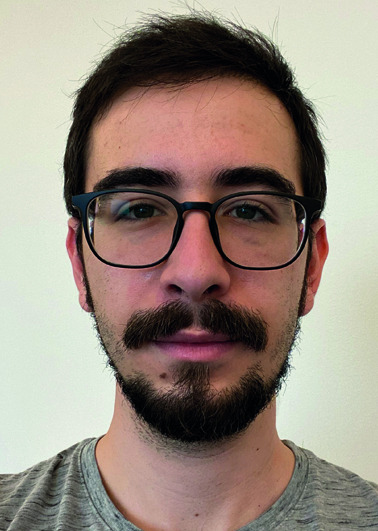

## Biographical Information

*Isidro M. Pastor earned his doctorate from the University of Alicante (Spain) in 2000. He completed a postdoctoral stay (2000–2002) with Prof. Hans Adolfsson at Stockholm University (Sweden), researching asymmetric catalysis. Returning to the University of Alicante, he conducted his teaching and research activities as Teaching Assistant (2003), Assistant Professor (2004), Lecturer (2007), Associate Professor (2008), and Professor (2021). His research, conducted within the “Alternative Methodologies in Chemistry” group, which he directs, focuses on sustainable chemistry, organometallic and heterocyclic chemistry, material chemistry ‐including metal‐organic structures‐ and catalysis*.

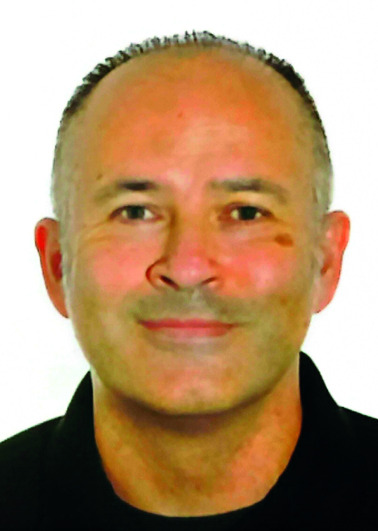

## Data Availability

Data sharing is not applicable to this article as no new data were created or analyzed in this study.
